# Identification of Novel Type 2 Diabetes Candidate Genes Involved in the Crosstalk between the Mitochondrial and the Insulin Signaling Systems

**DOI:** 10.1371/journal.pgen.1003046

**Published:** 2012-12-06

**Authors:** Josep M. Mercader, Montserrat Puiggros, Ayellet V. Segrè, Evarist Planet, Eleonora Sorianello, David Sebastian, Sergio Rodriguez-Cuenca, Vicent Ribas, Sílvia Bonàs-Guarch, Sorin Draghici, Chenjing Yang, Sílvia Mora, Antoni Vidal-Puig, Josée Dupuis, DIAGRAM Consortium, Jose C. Florez, MITIN Consortium, Antonio Zorzano, David Torrents

**Affiliations:** 1 Joint IRB–BSC Program on Computational Biology, Barcelona Supercomputing Center, Barcelona, Catalonia, Spain; 2 Computational Bioinformatics, National Institute of Bioinformatics, Madrid, Spain; 3 Center for Human Genetic Research and Department of Molecular Biology, Massachusetts General Hospital, Boston, Massachusetts, United States of America; 4 Program in Medical and Population Genetics, Broad Institute, Cambridge, Massachusetts, United States of America; 5 Biostatistics and Bioinformatics Unit, Institute for Research in Biomedicine, Barcelona, Spain; 6 Institute for Research in Biomedicine, Universitat de Barcelona, and CIBERDEM, Barcelona, Spain; 7 University of Cambridge, Metabolic Research Laboratories Institute of Metabolic Sciences, Addenbrooke's Hospital, Cambridge, United Kingdom; 8 Department of Computer Science, Department of Clinical and Translational Science, Department of Obstetrics and Gynecology, and Intelligent Systems and Bioinformatics Laboratory, Wayne State University, Detroit, Michigan, United States of America; 9 Institute of Translational Medicine, Cellular and Molecular Physiology, Liverpool, United Kingdom; 10 Department of Biostatistics, Boston University School of Public Health, Boston, Massachusetts, United States of America; 11 National Heart, Lung, and Blood Institute's Framingham Heart Study, Framingham, Massachusetts, United States of America; 12 Diabetes Unit, Center for Human Genetic Research and Diabetes Research Center, Massachusetts General Hospital, Boston, Massachusetts, United States of America; 13 Department of Medicine, Harvard Medical School, Boston, Massachusetts, United States of America; 14 Institució Catalana de Recerca i Estudis Avançats (ICREA) Barcelona, Spain; The University of North Carolina at Chapel Hill, United States of America

## Abstract

Type 2 Diabetes (T2D) is a highly prevalent chronic metabolic disease with strong co-morbidity with obesity and cardiovascular diseases. There is growing evidence supporting the notion that a crosstalk between mitochondria and the insulin signaling cascade could be involved in the etiology of T2D and insulin resistance. In this study we investigated the molecular basis of this crosstalk by using systems biology approaches. We combined, filtered, and interrogated different types of functional interaction data, such as direct protein–protein interactions, co-expression analyses, and metabolic and signaling dependencies. As a result, we constructed the mitochondria-insulin (MITIN) network, which highlights 286 genes as candidate functional linkers between these two systems. The results of internal gene expression analysis of three independent experimental models of mitochondria and insulin signaling perturbations further support the connecting roles of these genes. In addition, we further assessed whether these genes are involved in the etiology of T2D using the genome-wide association study meta-analysis from the DIAGRAM consortium, involving 8,130 T2D cases and 38,987 controls. We found modest enrichment of genes associated with T2D amongst our linker genes (p = 0.0549), including three already validated T2D SNPs and 15 additional SNPs, which, when combined, were collectively associated to increased fasting glucose levels according to MAGIC genome wide meta-analysis (p = 8.12×10^−5^). This study highlights the potential of combining systems biology, experimental, and genome-wide association data mining for identifying novel genes and related variants that increase vulnerability to complex diseases.

## Author Summary

It has been shown that the crosstalk between insulin signaling and the mitochondria may be involved in the etiology of type 2 diabetes. In order to characterize the molecular basis of this crosstalk, we mined and filtered several interaction databases of different natures, including protein–protein interactions, gene co-expression, signaling, and metabolic pathway interactions, to identify reliable direct and indirect interactions between insulin signaling cascade and mitochondria genes. This allowed us to identify 286 genes that are associated simultaneously with insulin signaling and mitochondrial genes and therefore could act as a molecular bridge between both systems. We performed in vitro and in vivo experiments where the insulin signaling or the mitochondrial function were disrupted, and we found deregulation of these connecting genes. Finally, we found that common variants in genomic regions where these genes lie are enriched for genetic associations with type 2 diabetes and glycemic traits according to large genome-wide association meta-analyses. In summary, we reconstructed the network implicated in the crosstalk between the mitochondria and the insulin signaling and provide a list of genes connecting both systems. We also propose new potential type 2 diabetes candidate genes.

## Introduction

Insulin resistance is a common trait present in complex disorders such as type 2 diabetes (T2D), obesity or metabolic syndrome (MetS). Around 340 million people suffer from diabetes worldwide, 90% of whom have T2D (http://www.who.int/diabetes/facts/en). Unlike type 1 diabetes, overt T2D is usually diagnosed several years after its onset due to its milder presenting symptoms, which in part explains why several devastating complications such as cardiovascular related diseases tend to develop soon after or have already arisen at the moment of the initial diagnosis.

There has been growing interest in identifying genes and processes that could trigger insulin resistance beyond defects on the insulin signaling cascade itself. As a result, defective mitochondrial activity has been indirectly related to insulin resistance in insulin-targeted tissues, such as skeletal muscle [1], [2], [3] and liver [4]. In particular, patients with T2D and, more importantly, non-diabetic subjects with type 2 diabetic relatives showed mitochondrial dysfunction and lower expression of PPAR gamma co-activator 1 alpha and 1 beta (PGC-1α and PGC1-1β), which are key regulators of mitochondrial biogenesis and function. In addition, subjects with early-onset type 2 diabetes typically show defective activation of PGC-1alpha in response to physical activity [5], and similarly, morbid obese type 2 diabetic patients show a defective activation of mitochondrial gene expression in response to weight-loss surgery [5]. Whether there is a heritable component involved in the alterations in expression of mitochondrial genes/proteins in these common forms of T2D remains to be determined.

Despite all of these efforts and lines of evidence, the mechanisms and the molecular contributors to the connection between mitochondria and the insulin signaling and resistance are still unknown. The availability of a wide range of functional interaction data, including metabolomics, genomics, transcriptomics and proteomics and the integration of all these data using systems biology approaches make it now possible to investigate in detail the molecular basis of the interaction between the insulin signaling cascade and mitochondrial biology in healthy and pathological scenarios, particularly in the context of T2D.

In addition, and despite substantial progress achieved in the identification of candidate genes involved in specific complex processes or diseases through genome-wide association studies (GWAS), for most diseases, including T2D, less than 10% of the heritability (percentage of variance attributable to genetic variation) can be explained by the identified genetic associations [6]. Some hypotheses suggest that a portion of the *missing heritability* stays behind multiple small effect size variants that have not yet reached genome-wide significance in GWAS meta-analyses when tested individually, due to insufficient sample sizes. If many of the modest effect variants are assumed to implicate genes that function in a limited number of biological processes, collective analysis of variants based on prior biological knowledge could substantially enhance association detection power. In that sense, the application of systems biology approaches to analyze GWAS data may have the potential to increase the chances of unraveling susceptibility genes or biological processes for complex diseases.

In this study, we applied systems biology approaches to screen and identify novel candidate T2D genes. The search has been guided by the hypothesis that the functional components of the crosstalk between the insulin signaling pathway and the biology of the mitochondria may play a role in the etiology or the evolution of the disease. We have also generated and analyzed gene expression data on insulin resistance and mitochondria perturbed scenarios to support these candidate genes. We finally tested whether particular genetic variants in loci that contain the identified genes could be collectively associated with T2D.

## Results

### Generation of the MITIN network

**Figure 1:**
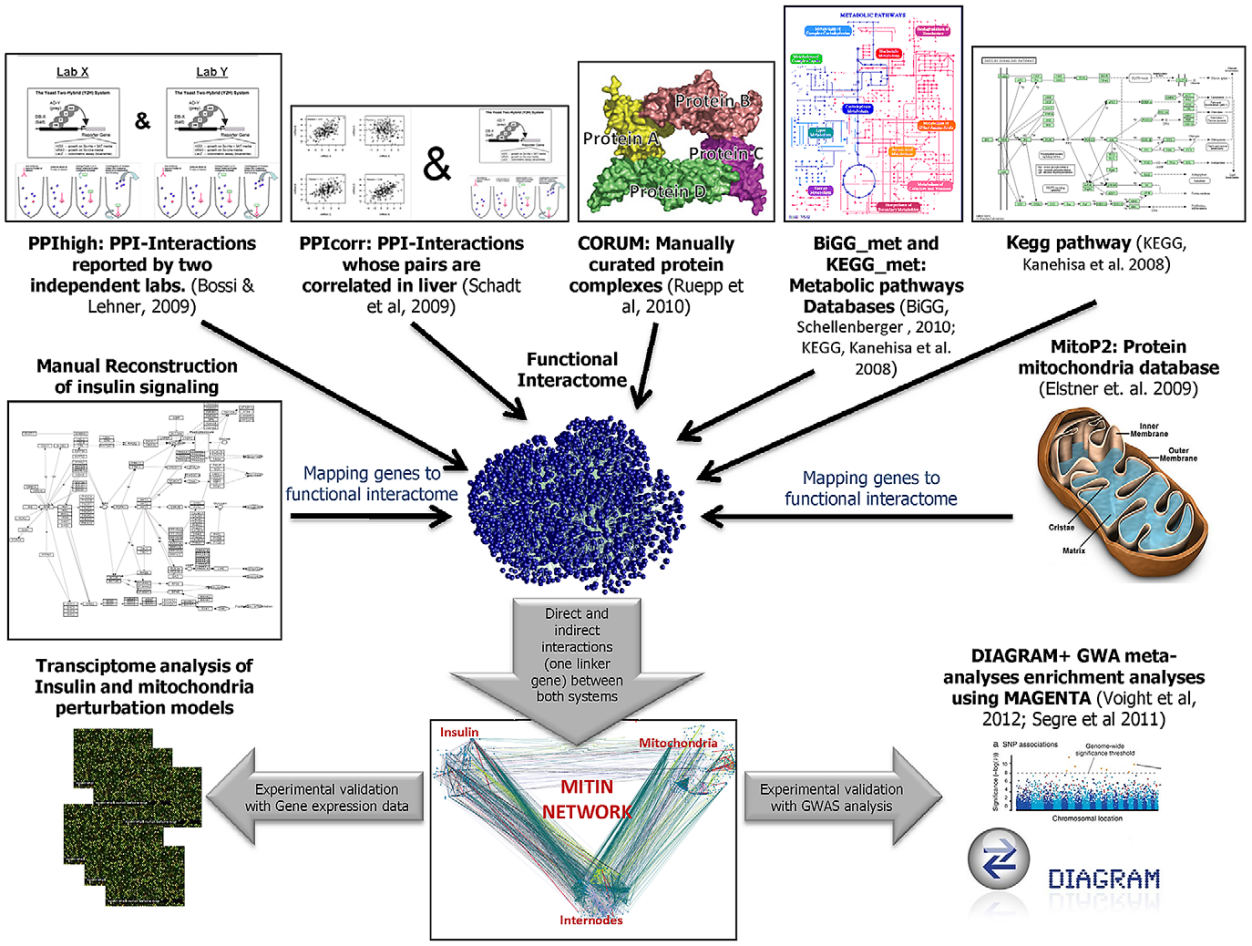
Schematic flow chart of the generation and evaluation of the MITIN network. The different sources of functional interaction are combined to generate a functional interactome. The resulting network is used to identify the direct and indirect interactions between the insulin signaling and mitochondria systems. The relevance of the MITIN network is tested analyzing gene expression data of models perturbing either insulin signaling or mitochondria function, and testing the variability within or near the MITIN network genes using GWA meta-analyses from DIAGRAM consortium. *In all PPIhigh and PPIcorr, both pair of interacting proteins have to be simultaneously expressed in any of the insulin-targeted tissues (adipose tissue, muscle, liver and heart).

In order to identify genes specifically involved in the crosstalk between the insulin signaling pathway and the mitochondria, we looked for all possible direct and indirect functional interactions between mitochondria and insulin signaling genes (Figure 1). We started by building reliable models and parts lists for these two systems. We first explored and manually filtered several public versions of the insulin signaling pathway to end up with a confident collection of 197 proteins/genes (see Methods). At the same time, we extracted data from a database of nuclear and mitochondrial-encoded mitochondrial proteins (MitoP2) to generate the corresponding list of 682 mitochondria genes [7].

Once both parts lists were constructed, we screened several large functional interaction databases to identify direct and indirect connections involving any of the protein/genes of each of the systems. We applied several filters and cutoffs to be able to isolate, from all available interactions, a reliable collection that will be used further in our study. For example, from protein-protein interaction (PPI) data, we only considered those protein pairs whose interactions were reported by two or more independent laboratories (PPIhigh) and whose pair of genes were reported to be expressed both in any of the insulin-sensitive tissues (adipose tissue, muscle, liver and heart, [8]); or any other PPI interaction reported only by a single laboratory, simultaneously expressed in any of the insulin-sensitive tissues and that also showed co-expression (gene-expression correlation) in a dataset of 427 healthy human liver samples [9] (these interactions are here termed PPIcorr). As a third layer of functional interaction, we also linked those proteins observed to belong to the same protein complex as described in the CORUM protein complex database [10]. The fourth source of interaction consisted of pairs of genes coding for enzymes that participate in linked metabolic reactions, i.e. those reactions that are adjacent in a metabolic reaction map according to the Biochemical Genetic and Genomic (BiGG_met) and the Kyoto Encyclopedia of Genes and Genomes database (http://www.genome.jp/kegg/kegg2.html; KEGG_met) [11], [12], [13]. Finally, we also included those interactions between genes coding for complexes or genes linked in a signaling pathway, as defined by KEGG (KEGG_path) [12]. This final functional interactome comprised 57,751 high confidence functional interactions involving 6963 genes, which represent a whole functional network of insulin-targeted tissues or cells.

From the pool of selected high quality interactions (affecting 6963 genes), we finally selected those interactions that, either directly or indirectly, provide a link between the mitochondrial and the insulin signaling cascade genes. We defined indirect interactions as those mediated by genes, termed linker or internode genes, that do not belong to either the insulin or the mitochondria parts list, but that are simultaneously connected to both systems. By applying these filters, we finally generated the mitochondria-insulin (MITIN) network consisting of 886 genes and a total of 1259 interactions, 70 direct (Table S1) and 1194 indirect. The 70 direct interactions involved 44 insulin genes and 37 mitochondria genes, most of them showing only one evidence of interaction. Both the insulin and mitochondria genes that were directly connected were linked to a median of two genes from the other system. Direct connections showed heterogeneous sources of interaction: PPIhigh, PPIcorr, Corum Complexes, BiGG_met, KEGG_met, Kegg_pathway, contributed 41, 9, 13, 2, 3, 12 links, respectively. Indirect interactions involved 286 linker internode genes (Figure S1, Dataset S1, Table S2 and S3). These internodes genes were connected to a mean number of 2.1 Insulin and 1.7 mitochondria genes and showed a mean of 2.6 and 2.0 lines of evidence of interaction with insulin and mitochondria, respectively. Regarding the 1194 indirect connections, PPI, PPIcorr, Corum Complexes, BiGG_met,KEGG_met, Kegg_pathway, contributed 570, 472, 1263, 42, 160, 169 interactions, respectively.

**Figure 2:**
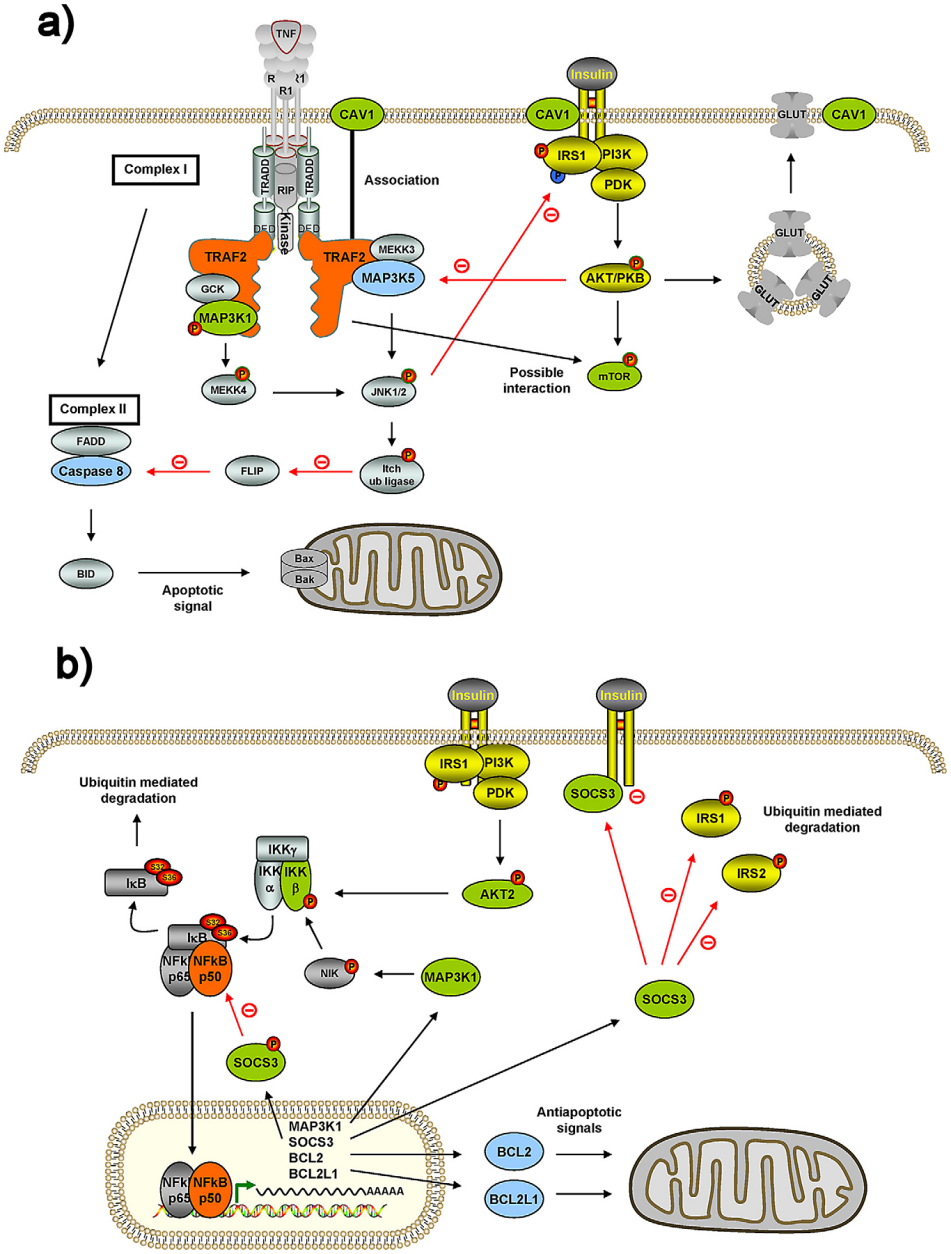
Connections of two internode genes, TRAF2 and NFKB1, with insulin genes and mitochondria genes. Two strong candidates linking both insulin and mitochondria genes from Table 1 were chosen and their connections to insulin genes and mitochondria genes verified using literature published in the PubMed. See main text for detailed description. A) TRAF2 has been reported to be connected to MAP3K1 (MEKK1) and CAV1 (caveolin 1) insulin genes, and to MAP3K5 (ASK1) and CASP8 (caspase-8) mitochondrial genes. A possible connection to MTOR (mTOR) has also to be considered. MAP3K5 = ASK1; MAP3K1 = MEKK1. B) NFKB1 (NF-κB1) is connected to IKBKB (IKKβ), AKT2, MAP3K1 and SOCS3 insulin genes, and BCL2 and BCL2L1 mitochondrial genes. NFKB1 = NFKBp50; IKBKB = IKKβ = IKK2; MAP3K1 = MEKK1; P65 = RelA. Green boxes represent insulin genes reported to interact with TRAF2 or NFKB1 according to our network; and light-blue represents mitochondrial genes reported to interact with TRAF2 or NFKB1 according to our network. Yellow boxes represent insulin signaling genes.

While the majority of the internode genes seem to be novel, as their bridging role connecting both systems has not yet been described, some of them have already been shown to interact with both systems, which constitutes an internal positive control of our underlying search methodology. For example, TRAF2 shows interactions within our MITIN network with four insulin and two mitochondrial genes (Table 1). Interestingly, other independent studies and approaches also identified five of these interactions. In particular with MAP3K1 (MEKK1), CAV1 (caveolin-1) and MTOR (mTOR), from the insulin signaling [14], [15], [16] and MAP3K5 (ASK1) and CASP8 (caspase-8) from the mitochondria [17], [18] (Figure 2). Another example is NFKB1, for which we found interactions with four insulin signaling and three mitochondrial genes. As above, NFKB1 has been also reported to interact with the *IKBKB* [19], [20], *AKT2* [21], *MAP3K1* [22] and *SOCS3* insulin genes, as well as to *BCL2L1* [22] [23] and *BCL2* [24] (Figure 2).

**Table 1:** Strong candidates linking both insulin and mitochondria genes.

Internode gene	Total links	Total evidences	Insulin associated genes	Evidences linking to insulin signaling	Mitochondria associated genes	Evidences linking to mitochondria	Insulin functional associations	Mitochondria functional associations
**ABL1**	12	21	10	18	2	3	YWHAH | CBL | SRC | CRK | BCR | PTK2 | CRKL | GRB2 | CSK | NCK1	WASF1 | TP53
**ALDOA**	6	8	2	4	4	4	FBP1 | FBP2	ALDH1B1 | ALDH1A3 | ALDH2 | GPD2
**ALDOB**	5	7	2	4	3	3	FBP1 | FBP2	ALDH1A3 | ALDH1B1 | ALDH2
**ALDOC**	5	7	2	4	3	3	FBP2 | FBP1	ALDH1B1 |ALDH1A3 | ALDH2
**DHX9**	5	6	2	3	3	3	NOLC1 | RPS6	BRCA1 | SLC25A5 | TUFM
**HNRNPU**	6	6	3	3	3	3	NOLC1 | YWHAQ | RPS6	C1QBP | SLC25A5 | TUFM
**HSP90AA1**	13	16	9	11	4	5	CALM1 | YWHAG | YWHAH | IKBKB | SRC | YWHAB | PDPK1 | YWHAQ | AKT1	TP53 | TOMM34 | TOMM70A | HSPD1
**ILF3**	6	6	3	3	3	3	NOLC1 | RPS6 | YWHAQ	SLC25A5 | C1QBP | TUFM
**MYBBP1A**	6	8	3	4	3	4	PKLR | RPS6 | NOLC1	POLG | SLC25A5 | TUFM
**NCL**	7	10	4	6	3	4	RPS6 | PRKCZ | PPARGC1A | NOLC1	TUFM | TP53 | SLC25A5
**NFKB1** #	7	9	4	6	3	3	IKBKB | SOCS3 | MAP3K1 |AKT2	BCL2L1 | BCL2 |MTIF2
**NFKBIA**	5	8	3	5	2	3	SRC | CAPN1 | IKBKB	TP53 | SLC25A5
**NOS1** *	8	9	3	3	5	6	HRAS | PRKACA | CALML3	SLC25A15 | SLC25A2 | OTC | ASS1 | PRKCA
**NOS3**	6	8	2	3	4	5	CAV1 | AKT1	SLC25A2 | ASS1 |SLC25A15 | OTC
**NPM1**	6	7	3	3	3	4	RPS6 | GRB2 | NOLC1	TUFM | SLC25A5 | TP53
**PKM2**	4	6	1	3	3	3	PKLR	POLG |AK3L1 | AK2
**PRNP**	6	7	3	4	3	3	CAV1 | MAP2K1 | GRB2	HSPD1 | BAX | SOD1
**RB1**	8	9	3	3	5	6	INS | MAPK1 | PIK3R3	C1QBP |BRCA1 | TGM2 | PHB | TRAP1
**RELA**	7	10	4	7	3	3	IKBKB | CALM1 | IKBKB | PRKCZ	ETHE1 | MTIF2 | ESR1
**RPL10**	5	6	2	3	3	3	RPS6 | NOLC1	PRKCA | SLC25A5 | TUFM
**RPL11**	4	6	2	3	2	3	RPS6 | NOLC1	SLC25A5 | TUFM
**RPL30**	5	6	2	3	3	3	RPS6 | NOLC1	TUFM | SLC25A5 | MRPS15
**RPL35A**	5	6	2	3	3	3	NOLC1 | RPS6	SLC25A5 | TUFM | MPG
**RPS14**	4	6	2	3	2	3	RPS6 | NOLC1	TUFM | SLC25A5
**RPS9**	5	6	2	3	3	3	NOLC1 | RPS6	TUFM | SLC25A5 | TOMM40
**SNRPD1**	7	7	4	4	3	3	EIF4E | PPP1CA | YWHAB | YWHAQ	C1QBP | HSPD1 | TRAP1
**TOP1**	5	6	2	3	3	3	RPS6 | NOLC1	TUFM | SLC25A5 | TP53
**TRAF2**	6	9	4	6	2	3	MAP3K1 | CAV1 | MAPK10 | MTOR	CASP8 | MAP3K5
**TRAF6**	9	12	5	7	4	5	PRKCZ | SRC | MAP3K1 | MAPK10 | MAP2K1	NDUFA1 | MT-CO2 | HADHA | NDUFAF1
**U2AF1**	6	7	2	3	4	4	NOLC1 | RPS6	SLC25A5 | TRAP1 | TUFM | HSPD1
**YBX1**	6	8	3	3	3	5	RPS6 | NOLC1 | AKT1	SLC25A5 | TUFM | TP53

The internode genes listed in the table have at least three lines of evidence that link them to the mitochondria and three to insulin signaling.

# Above 95% percentile of T2D association gene scores based on DIAGRAM meta-analysis (see Table 2).

* Associated to HOMA-IR(9.74E–6) [42].

The same MITIN network also allowed us to define which mitochondrial genes are more connected to insulin signaling, and *vice-versa*, either directly or indirectly. The top five insulin signaling genes most connected to mitochondria are *NOLC1*, *RPS6*, *IKBKB*, *PKLR*, *SRC*, with a total of 99, 40, 31, 28 and 22 indirect connections with mitochondria, respectively. Similarly, the five most connected mitochondrial genes with the insulin cascade were *TUFM*, *TP53*, *SLC25A5*, *POLG*, *ESR1*, with a total of 93, 36, 29, 25, and 19 indirect connections, respectively (Table S4).

We next explored whether our collection of internode genes where enriched in particular functions or processes by querying the Molecular Signatures Database [25]. We found up to 148 functional signatures for which internode genes were significantly enriched (5.7×10^−107^<p value<4.41×10^−6^, 1.94<Odds ratio<20.1; Table S5). Besides several enriched categories related to translation, *Reactome Regulation of Expression in Beta Cells* (p = 3.5×10^−87^, Odds Ratio = 15.8), *Reactome Insulin Synthesis and Secretion* (p = 4.46×10^−79^, Odds ratio = 14.0), and *Reactome Diabetes pathways* (p = 1.39×10^−35^, Odds ratio = 5.5) were also highly enriched among our set of internode genes. No significant categories were found after correcting for multiple testing in a set of internode genes identified from a simulated network made of randomly generated interactions.

In order to facilitate the selection of any of these genes for further studies, we have ranked them according to their number of connections to each of the systems. Hence, we provide a confident subset of 31 genes with at least three lines of evidence linking insulin signaling and mitochondria genes simultaneously (Table 1).

### Internode gene expression is altered in insulin resistance and mitochondrial dysfunction experimental models

As further support of the functional relationship between internode genes and both, the mitochondria and the insulin signaling pathway, we explored whether the expression of these identified internode genes is modified after perturbing each of the mitochondria or insulin signaling systems independently.

**Figure 3:**
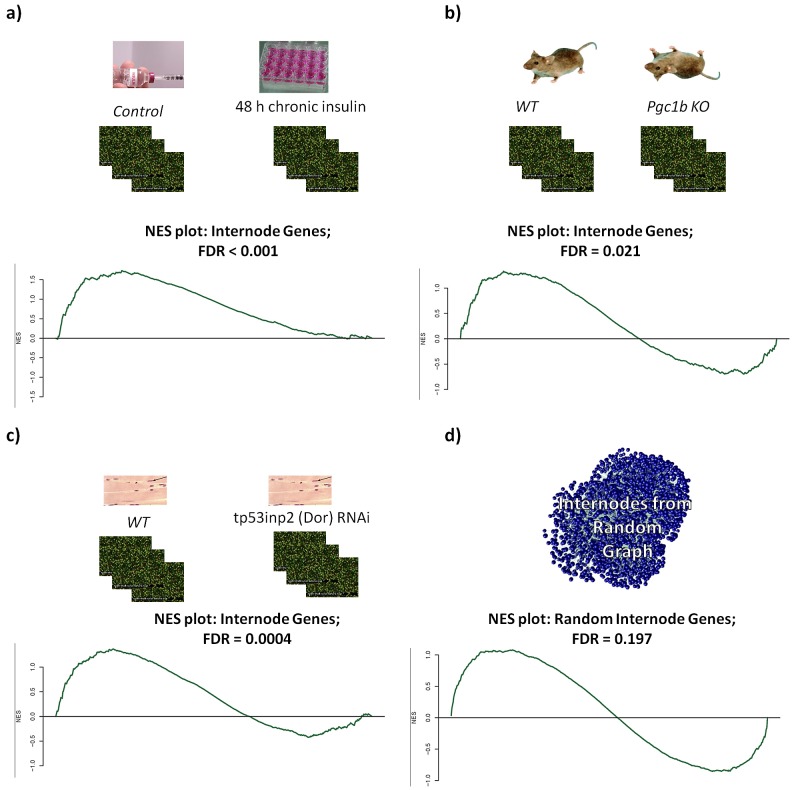
Gene set enrichment analysis. Gene set enrichment analysis of models with impaired Insulin (a, c) or mitochondrial (b) function. In all cases there was enrichment of upregulated genes within the internodes, except for the case when internodes were generated from a random network (d).

To test the effect of the insulin signaling perturbation, we performed gene expression profiling of C2C12 differentiated myotubes that were either left untreated or treated with 100 nM insulin for 2 days in order to induce an insulin resistance state. This treatment resulted in the downregulation of the insulin receptor and subsequently significantly reduced insulin signaling cascades [26]. We used the gene set enrichment analysis method (GSEA, [25]) to look for enrichment of differential expression using our set of internode, mitochondria, and insulin genes as molecular signatures. Using the collection of all 6963 genes with identified interactions as a background, we found significant enrichment of upregulation within the internode genes (Normalized Enrichment Score (NES) = 1.7; False Discovery Rate (FDR) = 0.0013), while observed downregulation enrichment within the insulin signaling genes (NES = −1.4; FDR = 0.028) (Figure 3a). We also explored a second model of insulin signaling cascade perturbation through the analysis of transcriptome data from myotubes treated with RNAi against DOR (also named *Tp53inp2*). This gene is dysregulated in muscle of Zucker diabetic rats, participates in the myogenic differentiation and mediates a feed-forward loop between ecdysone receptor and the insulin signaling in flies [27], [28]. In this model, we also found that there was an enrichment of upregulated internode genes (NES = 1.4; FDR = 0.004) and enrichment of downregulated insulin (NES = −1.35; FDR = 0.007) and mitochondrial (NES = −1.36; FDR = 0.001) genes (Figure 3c).

In a parallel experiment we tested how perturbations of mitochondria affect the expression of the MITIN network genes. For this, we analyzed gene expression from the heart of Peroxisome-proliferator-activated-receptor γ coactivator 1 beta (PGC-1β) knock-out mice. PGC-1β is a co-activator that regulates mitochondrial biogenesis and function [29], [30], [31], [32]. The analysis of heart gene expression of these mice showed an overrepresentation of upregulated genes within the internodes (NES = 1.3; FDR = 0.02), enrichment of upregulated genes within the insulin genes (NES = 1.6; FDR = 0.0012), and enrichment of downregulated mitochondria genes (NES = −2.63; FDR = <0.0001) (Figure 3b). Again, as a control from our experiment, randomly generated internode genes did not show any enrichment in any of these experiments (Figure 3d).

### Clinical implications of the Global MITIN Network

We next investigated whether any of these genes has been associated to phenotypes related to insulin resistance or energy metabolism. For this, we searched through the OMIM database (http://www.ncbi.nlm.nih.gov/omim) those internode genes that are involved in mendelian and complex disorders [33].

We found that, among all 286 internode genes, 191 (66%) were in genomic loci associated to complex diseases or traits (SNPs within 250 kb from internode gene were considered) and 17 (6%) were involved in mendelian diseases. Interestingly 53 of the genes (18%) contained or were near polymorphisms associated to T2D or related traits such as obesity, adiposity, response to glucose challenge, hypertension or coronary artery disease (Table S6). 10,000 random simulations showed that finding 53 genes associated to T2D related traits was modestly more than what expected by chance (p = 0.0535). In contrast, the 10,000 random simulations also showed that we did not find more associations with any complex trait (not restricting to T2D related traits), than would be expected by chance, suggesting that the enrichment for associations of the identified internode genes is specific for T2D and related metabolic traits.

### Scanning T2D genome-wide association meta-analyses for variants in the internode genomic regions shows enrichment of T2D associations

In order to further investigate the potential involvement of the internode genes in the etiology of T2D, we screened the DIAGRAM consortium GWAS dataset, which consisted on the largest T2D meta-analysis available at the time of the study (DIAGRAM meta-analysis): 8,130 cases and 38,987 controls [34]. To analyze enrichment of associated genes within the internodes, we used MAGENTA [35], a software specifically designed for large genome-wide association study meta-analyses, where individual genotypes are typically not available. We found that our internode gene list showed nominal enrichment for modest to strongly associated genes within the top 5% of T2D scores, with 18 genes observed, including three already confirmed T2D associated SNPs [34], [36], [37], compared to the 12 expected by chance (p = 0.0549, Table 2). These results were robust to the enrichment cutoff used (p = 0.0368 when testing for enrichment above the 97.5th percentile of all gene scores; 6 genes expected above cutoff, 11 observed). Unlike the collection of internode genes, no significant enrichment for T2D associations was found for gene-sets belonging only to the insulin signaling (p = 0.71) or to the mitochondrial (p = 0.52) systems. The insulin and mitochondria genes directly interacting with each other were also not enriched for T2D associations (p = 0.53).

**Table 2:** Internode genes that fall in the 95% percentile of T2D association gene scores based on DIAGRAM meta-analysis using MAGENTA, and putative associations with T2D-related traits.

HGCN Name	Gene p-value	Chr	Best local SNP	Best SNP Chr Pos (bp)**	Best SNP pval	Best SNP OR	Distance from gene (Kb)	Previously reported T2D associations	Other quantitative associated Traits (Trait: rsid; p value; GeneLoc; distance in Kb)
NFKB1#	1.26E-05	4	rs7674212	104.208	1.70E-07	1.114	450.8		**DBP:** rs13107325; 7.53e-07; UP; 233.8 | **BMI:** rs13107325; 1.37e-07; UP; 233.8 | **HDL:** rs13107325; 7.2e-11; UP; 233.8 | **SBP:** rs13107325; 2.57e-07; UP; 233.8
IQGAP2	1.66E-05	5	rs4457053	76.461	4.15E-08	0.8607	421.0	[34]	**HbA1C:** rs6453220; 4.19e-06; inGene; 0
RPL32	1.31E-03	3	rs6802898	12.366	3.18E-06	1.1537	485.2		**LDL:** rs11709504; 2.15e-07; UP; 201.8 | **TC:** rs2290159; 4.21e-09; UP; 247.1 | **TG:** rs17819328; 5.09e-07; UP; 386.7
IQGAP1*	3.03E-03	15	rs8042680	89.322	8.19E-06	1.1018	475.9	[34]	**DBP:** rs2521501; 6.06e-07; DW; 391.9 | **SBP:** rs2521501; 1.22e-07; DW; 391.9
RPS13	6.03E-03	11	rs5215	17.365	1.60E-05	1.0934	309.4	[36], [37]	**DBP:** rs381815; 1.22e-07; UP; 193.7 | **SBP:** rs381815; 2.45e-09; UP; 193.7
LRPPRC	9.98E-03	2	rs11899863	43.472	1.04E-05	1.1688	494.6		**LDL:** rs4299376; 1.73e-47; UP; 40.8 | **FastGluc:** rs11899863; 2.58e-06; UP; 494.5 | **HOMA-B:** rs10203174; 9.8e-06; UP; 423.3 | **SBP:** rs10206724; 5.57e-06; inGene; 0 | **TC:** rs4299376; 4.03e-45; UP; 40.8
LSM8	1.04E-02	7	rs10244364	117.317	2.97E-05	1.0916	294.4		
CD3EAP	1.06E-02	19	rs4420638	50.115	5.41E-05	1.1468	486.5		**BMI:** rs2287019; 3.18e-07; DW; 288.1 | **HDL:** rs4420638; 4.4e-21; UP; 486.6 | **LDL:** rs4420638; 8.72e-147; UP; 486.6 | **2 hrGluc:** rs10423928; 3.33e-06; DW; 268.3 | **TC:** rs4420638; 5.2e-111; UP; 486.6 | **TG:** rs439401; 1.14e-30; UP; 495.0
KPNA2	1.08E-02	17	rs11655081	63.894	5.55E-05	1.2423	420.4		
PPP3CA	1.10E-02	4	rs1426538	102.669	1.99E-05	0.914	181.6		
POLH	1.58E-02	6	rs6933958	43.932	5.13E-05	1.1031	235.4		**WHR:** rs1358980; 1.38e-10; DW; 177.8 | **SBP:** rs10948071; 5.89e-06; UP; 263.2 | **TG:** rs998584; 4.06e-07; DW; 171.2
BCAR1	2.48E-02	16	rs9927309	73.804	6.61E-05	1.1388	16.5		
RAB4A	2.73E-02	1	rs3767331	227.831	6.53E-05	0.8521	323.9		
SRSF1	3.15E-02	17	rs886929	53.156	6.76E-05	1.0941	277.6		
GRM5	3.24E-02	11	rs16913724	87.621	2.70E-05	1.1309	259.7		
RPL22	3.67E-02	1	rs7368256	6.558	1.31E-04	1.1085	375.5		
H1FX	3.79E-02	3	rs9847824	130.8	2.52E-04	1.1309	282.5		**WHR:** rs2301573; 3.68e-06; DW; 270.8
RPL10A	4.67E-02	6	rs10484578	35.354	1.62E-04	1.0865	189.9		**HDL:** rs7742443; 6.27e-06; UP; 427.5 | **LDL:** rs3800406; 2.89e-07; UP; 303.1 | **FastIns:** rs13210323; 6.32e-06; UP; 431.1 | **TC:** rs3800406; 7.31e-09; UP; 303.1

Associations were looked-up in GWAS meta-analyses from the MAGIC, GIANT and ICBP consortiums for SNPs within 500 kb from the internode gene boundaries.

* upregulated in our chronic insulin treatment (fold change = 1.4; FDR = 0.004) and in the Dor RNAi experiment (fold change = 1.33; FDR = 0.1).

** Chromosome position based on genome build 36 (hg18).

# Within the high confidence internode set.

BMI: Body Mass Index; HDL: High Density Lipoprotein; LDL: Low Density Lipoprotein; 2 hrGluc: 2 hours glucose challenge; TC: total Cholesterol; WHR: Waist to Hip Ratio; TG: Triglycerides; DBP:Diastolic Blood Pressure; SBP: Systolic Blood Pressure; HbA1C: Glycaeted Hemoglobin; FastGluc: Fasting Glucose levels; HGNC: HUGO Gene Nomenclature committee.

To further support the involvement of at least some of these 18 internode SNPs in glucose metabolism regulation, we also computed how the best associated SNPs in the 18 regions increased the risk of altered glycemic traits, available from MAGIC consortium datasets [38], [39], [40], [41], [42], using an approximation approach developed by Toby Johnson [43]. Among the seven traits tested, we found a significant association risk score for fasting glucose (p = 8.12×10^−5^ including the 18 top ranked SNPs and p = 0.004 including 15 out of the 18 SNPs not previously associated with T2D). In order to evaluate the probability of finding such a highly statistical p-value, when using the top T2D associated genes (and best local SNPs) we ran MAGENTA on 10,000 simulated random gene-sets, and extracted for each simulation the p-values of the most significant SNP per gene for all genes that ranked above the 95th percentile. The empirical p-value was then calculated as the frequency of random gene-sets whose p-values were smaller than the one obtained with the real data and whose effect size was higher than 0. We found that 8.12×10^−5^ is significantly lower than what one can expect by chance (p = 0.0144), confirming the association of our set of internode genes, not only with T2D, but also to fasting glucose levels.

### Genetic variants in internode genomic regions associated with T2D are also associated with metabolic related quantitative traits

To further explore the involvement of the internode genes associated with T2D (see above) in related metabolic traits we explored several available GWA meta-analyses pertaining to obesity-related traits from the GIANT consortium [44], [45], seven glycemic traits from MAGIC datasets [38], [39], [40], [41], [42], and cardiovascular disease traits from the ICBP consortium [46]. We found that in 10 of the 18 internode genomic loci with modest to strong associations, there was at least one SNP showing association (p<10^−5^) to one of these metabolic traits. For example, rs6453220, located in the *IQGAP2* intron, was associated to circulating glycated hemoglobin (p = 4.19×10^−6^) and rs13107325, located upstream of *NFKB1*, was strongly associated with diastolic blood pressure (p = 7.53×10^−7^), body mass index (p = 1.37×10^−7^), high density lipoprotein levels (p = 7.2×10^−11^), and systolic blood pressure (p = 2.57×10^−7^).

## Discussion

Understanding the molecular basis of insulin resistance is essential for the early diagnosis, treatment and prevention of T2D and related co-morbidities, such as hyperlipidemia or cardiovascular disease. In this study we explored the molecular basis of insulin resistance beyond the known role of insulin signaling genes, and, implicitly screened for novel candidate T2D genes. Based on published evidence that connects the function of the mitochondria with insulin resistance and T2D [5], [47], [48], [49], we hypothesized that there are genes responsible for the crosstalk between the mitochondria and the insulin signaling system, which makes them good candidates for T2D. By screening and filtering a variety of available functional interaction data, we have first generated a conservative network (MITIN) containing all genes involved in or connected to the insulin signaling or mitochondrial systems, not only through PPI but also based on interactions of other nature, including co-expression, protein complexes, and signaling and metabolic interactions. From there, we then selected a fraction of 286 internode genes that show connections to genes of both systems and are, therefore, likely to be involved in the functional crosstalk between the insulin signaling cascade and the mitochondria.

We have examined these genes at different levels to validate their bridging role and their potential implication in T2D or co-morbidities. In order to provide a more stringent list amenable to low throughput molecular biology experiments in future studies on insulin resistance and diabetes, we ranked these genes on the basis of their level of connectivity to insulin and mitochondrial genes and generated a high confidence subset of 31 genes showing three or more functional connections to each of the systems.

While there are no reported confirmatory data for the majority of the 286 internode genes, some have been already found to be linked to both systems, and even to T2D and related metabolic processes. For example *TRAF2* [14], [15], [17], [18], *NFKB1* [19], [20], [21], [22], [23], [24] (Figure 2) and *SMAD3* [50], which show multiple connections to insulin signaling and to mitochondrial genes in our MITIN network, have also been described elsewhere to interact with genes of both systems. In addition, variants near the *NFKB1* gene have been associated to T2D based on the DIAGRAM dataset (best nearby SNP p-value = 1.6×10^−5^), while *SMAD3* has been recently found to protect against diet-induced obesity as well as coronary artery disease [50], [51]. Other genes that also emerge as connecting internode genes in our MITIN network, such as the chaperone *HPSP90AA* gene, have not been previously described as linked to the insulin or the mitochondrial systems, but have been linked to insulin resistance conditions and hence to T2D [52], [53].

On top of the previous knowledge on some of the internode genes, we provide here further evidence that supports the robustness of our search strategy and of this collection of genes as potential molecular connectors of these systems, as well as insulin resistance or T2D candidate genes. First, the 286 internode genes showed significant enrichment of functional categories, like “*regulation of beta cell development*” *(p = 2.1×10^−79^)*, “*insulin synthesis and secretion*” *(p = 3.4×10^−79^)* and “*diabetes pathways*” (p = 1.9×10^−35^). Second, experimental models of mitochondria and insulin signaling perturbation caused a significant upregulation of the internode genes. This could be the result of direct regulation or a mechanism that compensates these perturbed metabolic scenarios. In all cases, the expression analyses helped us to confirm that these genes are indeed functionally connected to both systems. Furthermore, the deregulation of these internode genes under experimental conditions of insulin resistance suggests their involvement in T2D.

Encouraged by our positive functional and expression results supporting the connecting role of the internode genes and their impact on T2D, we went one step further and used the MITIN network as a basis for the identification of genetic signatures associated with T2D, contributing to unraveling its *missing heritability*. We tested for enrichment of T2D associations within the newly identified internode genes, by analyzing the results from the DIAGRAM GWA meta-analyses [34] using MAGENTA to define gene association scores and enrichment of gene associations [35]. We found enrichment of T2D variants within this group of genes, involving 18 associated genes compared to the 12 that were expected by chance (p = 0.0549). Our study also confirms the absence of significant signal when we tested insulin signaling and mitochondria gene-sets for enrichment of T2D associations. This is in agreement with previous studies, where no enrichment was found for mitochondrial or insulin signaling genes [34], [35], and suggests that the genes involved on the crosstalk between the insulin and mitochondria networks are more susceptible to harbor T2D risk variants than those that belong to either the insulin cascade or the mitochondria alone. The best local SNP in each of the 18 top ranked regions showed a combined risk score of increased fasting glucose levels according to MAGIC consortium data-sets (p = 8.12×10^−5^). Also supporting these results, several variants in the internode genomic regions identified by MAGENTA were also associated with many metabolic related quantitative traits, as reported by the MAGIC [38], [39], [40], [41], [42], GIANT [44], [45] and ICBP [46] consortia (Table 2).

Interestingly, the best-associated SNP in four of the 18 genes were among the 43 already validated loci of susceptibility for T2D, which in the former reports were assigned to *ZBED3*, *BCL11A*, *PRC1*, and *KCNJ11* genes, based only in proximity [34], [36], [37]. Taking into account the intrinsic challenge in linking an associated variant to its causal gene, we cannot exclude that these SNPs may be proxies for causal variants affecting our group of identified internode genes. Accordingly, recent findings suggest that a fraction of regulatory variants can be more than 500 Kb away from their regulated gene and that a single locus can expand more than 1 Mb, and even contain more than one independent causal variant [54], [55], [56]. Among the top 18 top ranked internode genes identified by MAGENTA analyses of T2D GWAS meta-analysis, there are independent lines of evidence suggesting the involvement on the development of T2D or insulin resistance. For example, two members of the IQ-motif-containing GTPase-activating protein (IQGAP) family, scaffold proteins involved in a wide range of cellular and signaling processes, including cytoskeletal organization, cell adhesion, and tumorigenic processes [57], [58], appear in the top 95th percentile for association with T2D according to MAGENTA analysis. IQ motif containing GTPase activating protein 2 (*IQGAP2*), the second ranked gene according to MAGENTA analysis, contained an intronic low frequency SNP (rs6453220; MAF = 0.05), which was strongly associated with glycated haemoglobin according to MAGIC WGA-meta-analyses (Hb1Ac; p = 4.19×10^−6^), providing more evidence that variants in *IQGAP2* may contribute to insulin resistance. In addition, another gene of the same family, *IQGAP1* (top four according to MAGENTA), was recently reported to bind the target of rapamycin complex 1 (mTORC1) having a potential negative feedback loop role upstream mTORC1/S6K1 AKT1 activation [59]. Furthermore, IQGAP1 associates with PKA and AKAP79 in pancreatic Beta cells, suggesting a role in the Beta-cell development and physiology [60]. It is also worth mentioning that *IQGAP1* was also found upregulated in our chronic insulin treatment experiment (fold change 1.4; FDR<0.01) and the *Tp53inp2* RNAi treatment in myotubes experiment (fold change = 1.33; FDR = 0.1). These results, together with the general role of scaffolding proteins as hubs of signaling pathways further supports the implication of the IQGAP protein family in the insulin signaling and the mitochondrial systems crosstalk and its association to T2D. *RAB4A* (Best SNP p value = 3.5×10^−5^) is a GTPase that regulates glucose transporter GLUT4 [61], and is suggested to participate in metabolic remodeling in the diabetic heart [62]. Finally, breast cancer anti-estrogen resistance 1 (*BCAR1*), (Best SNP p value = 6.61×10^−5^, distance from gene = 16.5 Kb) is another gene that deserves attention, as is connected to 10 insulin genes, according to our network: *CRK*, *SRC*, *PTPN1*, *PTK2*, *CRKL*, *PIK3R1*, *GRB2*, *PTPRF*, *RHOA* and *PTPRA*. Interestingly, a SNP in an intronic region 16 Kb upstream this gene was reported to be strongly associated with type 1 diabetes [63].

In summary, this study contributes to untangling the molecular basis linking the mitochondria and the insulin signaling systems and provides a subset of novel T2D candidate genes for further genetic, molecular and clinical studies. This study also constitutes a proof of concept of the utility of combining several integrative systems biology approaches with the analysis of gene expression and large GWA meta-analyses to uncover novel associations with complex diseases of otherwise hidden candidate genes.

## Materials and Methods

### Mitochondria and insulin parts lists

We constructed a consensus insulin pathway from several public resources, including (Biocarta; www.biocarta.com, Kegg [12]; www.genome.jp/kegg/, and PID; [64]; http://pid.nci.nih.gov/) and a commercial resource (Biobase; www.biobase.de). This pathway was manually curated and refined by the participation of molecular biologists in the field.

In order to select the parts lists that compose mitochondrial proteins or genes, we have selected a total set of 900 proteins from the mitoP2 database (www.mitop2.de/; [7]). As it was done for the insulin pathway, the set has been manually curated by the participation of the expert groups in the consortium.

To allow for transferability of the results to other species, we have identified each mouse orthologous gene/protein for all involved proteins.

### Generation of the MITIN network

To identify protein-protein interactions we used a non-redundant set of 23 protein interaction datasets and only included those interactions reported independently by two different laboratories (PPIhigh) [8].

For the gene co-expression analysis, we used the dataset of Schadt et al. [9], which consists of expression data of 427 healthy human liver samples and constituted the largest insulin-sensitive human transcriptome dataset. We evaluated the overlap between gene co-expression in liver and low confident PPIs (those reported only by a single lab) to provide a new source of high confident interactions.

Third, we added those interactions that pertained to the CORUM complex database [10], considering that two genes are functionally linked if they both pertain to a common complex.

The fourth source of interaction consisted of pairs of genes coding for those enzymes that participate in linked (or consecutive) metabolic reactions as described in KEGG or BiGG databases [11], [12], [13].

Finally, we also considered those interactions between genes coding for complexes or genes linked any signaling pathway, as defined by KEGG [12].

### Identification of enriched signatures

We used the Molecular Signatures Database from the Broad Institute ([25]; http://www.broadinstitute.org/gsea/msigdb) and for a total of 6770 gene sets, we computed an enrichment score based on a Chi-Square test. The corrected significant p-value after applying Bonferroni's correction for all the tests was 4.41×10^−6^. We only considered gene sets that had at least 10 genes within the group of internode genes.

### Microarray data analysis and GSEA

All statistical analyses were performed using Bioconductor (Gentleman et al., 2004). Microarray data was normalized via quantile normalization and summarized to probeset expression estimates via robust multi-array average (RMA) (Irizarry et al., 2003) using the function rma from the oligo package. All the newly generated data was deposited in the Gene Expression Omnibus (GEO) (http://www.ncbi.nlm.nih.gov/geo) database (GSE3932).

We used gene set enrichment analysis (GSEA) (Subramanian et al., 2005) as implemented in the Bioconductor library phenoTest [65] to assess the degree of association between gene expression and the following signatures: insulin, mitochondria and internodes. As indicated in Subramanian et al. [25], P-values were computed restricting attention to simulated ES with the same sign as ES_obs_.

### Chronic insulin treatment

All chemicals and reagents were purchased from Sigma-Aldrich, (Poole, UK). Briefly, C2C12 cells were cultured in Dulbecco's modified Eagle media (DMEM) supplemented with 10% Fetal bovine serum, and penicillin/streptomycin. To induce differentiation media was replenished by DMEM containing 2% (v/v) of horse serum with penicillin/streptomycin. Myotubes between days 4 and 7 following the induction of differentiation were used for experiments. For chronic insulin treatment cells were either left untreated or incubated with 100 nMinsulin in DMEM for 48 h in fusion medium to induce an insulin resistance state. Medium was changed every 24 h.

### Pgc1b knock-out model

Hearts were quickly collected and snap frozen in liquid nitrogen from wild-type and PGC-1β KO on a mix background (sv129 and C57BL/6) generated as previously reported [31]. Animal procedures were performed in accordance with the UK Home Office regulations and the UK Animal Scientific Procedures Act [A(sp)A 1986]. Animals were housed in a temperature-controlled room with a 12-h light/dark cycle. Food and water were available ad libitum.

### Dor silencing

Lentiviruses encoding scrambled or DOR siRNA were used as reported [27]. Fifteen million C2C12 myoblasts grown on 12-well plates were transduced at moi 100 and cells were amplified during 5–7 days. Transduced cells (GFP-positive) were then sorted with a MoFlo flow cytometer (DakoCytomation, Summit v 3.1 software), obtaining between 93%–99% GFP-positive cells. Confluent C2C12 myoblasts previously infected with lentiviruses encoding scrambled RNA or DOR siRNA were allowed to differentiate in 5% horse serum-containing medium for 4 days. Total RNA was purified and microarrays were performed by using an Affimetrix platform.

### Enrichment of T2D associations in internode genes

We used the latest DIAGRAM T2D GWA meta-analysis comprising 8,130 cases and 38,987 controls [34] and the MAGENTA software was used to test for enrichment of associations in the 286 internode genes [35]. Briefly, we assigned to each gene a set of SNPs that lie within 500 Kb upstream and downstream of the gene's most extreme transcript boundaries. This boundaries were based on the fact that a fraction of regulatory variants can be up to 500 Kb distal to their regulated gene and that a single locus may harbor more than one causal variants, and extend to more than 1 Mb from the locus top hit [54], [55], [56]. For each gene, a score was assigned based on the most significant SNP, followed by correction for confounders, including gene size, number of independent SNPs, and linkage disequilibrium-based properties. Once all the association scores were computed, MAGENTA tested for over-representation of genes in a given gene set above a predetermined gene score rank cutoff, which in this case was the 95th percentile of all gene scores. The enrichment is evaluated against a null distribution of gene sets of identical set size that were randomly sampled from the 6963 genes that constitute our complete interactome based on all identified functional interactions.

### Risk score analyses using multi–SNP predictors in glycemic traits from MAGIC consortium dataset

We computed how the best associated SNPs in the 18 regions could collectively increase the risk of altered glycemic traits available from MAGIC consortium datasets [38], [39], [40], [41], [42]. We used the method described in [43]. An unweighted genetic risk score was defined for each individual as the sum of the number of risk increasing alleles at each of the 18 SNPs of interest. If one had access to individual-level data, association between SNP score and glycemic traits could be tested using the usual approach. However, when the risk score involves SNPs in linkage equilibrium, it was shown [43] that association between risk score and trait can be assessed using meta-analysis results only, without going back to individual-level data. The effect of the risk score on the phenotype is estimated by
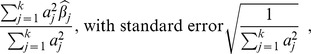
where 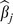 is the meta analysis effect size for SNP j, and a_j_ is the inverse of the standard error estimate of 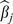.

The assumption of no Linkage Disequilibrium (LD) is required for the contribution of each SNP to be independent and for the standard error estimate to be valid. P-value for the risk score association can be assessed using the ratio of the SNP score effect estimate divided by its standard error, and assessing the significance of the ratio by comparing it to the standard normal distribution.

This large sample procedure will result in valid p-values under the null hypothesis of no relationship between the trait and variants included in the risk score.

## Supporting Information

Appendix S1 List of authors and affiliations of the MITIN and the DIAGRAM+ consortia.
(DOC)

Dataset S1 Cytoscape file of the MITIN network.
(ZIP)

Figure S1 Graphical representation of the MITIN network.
(TIF)

Table S1 Direct interactions between mitochondria and insulin cascade genes.
(XLS)

Table S2 Indirect interactions between mitochondria biology and insulin signaling cascade genes.
(XLS)

Table S3 Full list and description of internode genes.
(XLS)

Table S4 Number of direct and indirect connections of each mitochondria and in insulin gene to the insulin cascade and mitochondria systems, respectively.
(XLS)

Table S5 Functional enrichment of internodes in gene sets extracted from the Molecular Signatures Database.
(XLS)

Table S6 SNPs with distance less than 250 Kb to the internode genes associated with complex diseases or traits related to type 2 diabetes.
(DOC)

## References

[1] Petersen KF, Dufour S, Befroy D, Garcia R, Shulman GI (2004) Impaired mitochondrial activity in the insulin-resistant offspring of patients with type 2 diabetes. N Engl J Med 350: 664–671. 14960743 10.1056/NEJMoa031314 PMC2995502

[2] Petersen KF, Befroy D, Dufour S, Dziura J, Ariyan C, et al (2003) Mitochondrial dysfunction in the elderly: possible role in insulin resistance. Science 300: 1140–1142. 12750520 10.1126/science.1082889 PMC3004429

[3] Kelley DE, He J, Menshikova EV, Ritov VB (2002) Dysfunction of mitochondria in human skeletal muscle in type 2 diabetes. Diabetes 51: 2944–2950. 12351431 10.2337/diabetes.51.10.2944

[4] Lee HJ, Chung K, Lee H, Lee K, Lim JH, et al (2011) Downregulation of mitochondrial lon protease impairs mitochondrial function and causes hepatic insulin resistance in human liver SK-HEP-1 cells. Diabetologia 54: 1437–1446. 21347624 10.1007/s00125-011-2074-z

[5] Hernandez-Alvarez MI, Chiellini C, Manco M, Naon D, Liesa M, et al (2009) Genes involved in mitochondrial biogenesis/function are induced in response to bilio-pancreatic diversion in morbidly obese individuals with normal glucose tolerance but not in type 2 diabetic patients. Diabetologia 52: 1618–1627. 19504086 10.1007/s00125-009-1403-y

[6] Billings LK, Florez JC The genetics of type 2 diabetes: what have we learned from GWAS? Ann N Y Acad Sci 1212: 59–77. 10.1111/j.1749-6632.2010.05838.x PMC3057517 21091714

[7] Elstner M, Andreoli C, Klopstock T, Meitinger T, Prokisch H (2009) The mitochondrial proteome database: MitoP2. Methods Enzymol 457: 3–20. 19426859 10.1016/S0076-6879(09)05001-0

[8] Bossi A, Lehner B (2009) Tissue specificity and the human protein interaction network. Mol Syst Biol 5: 260. 19357639 10.1038/msb.2009.17 PMC2683721

[9] Schadt EE, Molony C, Chudin E, Hao K, Yang X, et al (2008) Mapping the genetic architecture of gene expression in human liver. PLoS Biol 6: e107 doi:10.1371/journal.pbio.0060107. 18462017 10.1371/journal.pbio.0060107 PMC2365981

[10] Ruepp A, Waegele B, Lechner M, Brauner B, Dunger-Kaltenbach I, et al (2009) CORUM: the comprehensive resource of mammalian protein complexes–2009. Nucleic Acids Res 38: D497–501. 19884131 10.1093/nar/gkp914 PMC2808912

[11] Schellenberger J, Park JO, Conrad TM, Palsson BO BiGG: a Biochemical Genetic and Genomic knowledgebase of large scale metabolic reconstructions. BMC Bioinformatics 11: 213. 20426874 10.1186/1471-2105-11-213 PMC2874806

[12] Kanehisa M, Araki M, Goto S, Hattori M, Hirakawa M, et al (2008) KEGG for linking genomes to life and the environment. Nucleic Acids Res 36: D480–484. 18077471 10.1093/nar/gkm882 PMC2238879

[13] Lee DS, Park J, Kay KA, Christakis NA, Oltvai ZN, et al (2008) The implications of human metabolic network topology for disease comorbidity. Proc Natl Acad Sci U S A 105: 9880–9885. 18599447 10.1073/pnas.0802208105 PMC2481357

[14] Feng X, Gaeta ML, Madge LA, Yang JH, Bradley JR, et al (2001) Caveolin-1 associates with TRAF2 to form a complex that is recruited to tumor necrosis factor receptors. J Biol Chem 276: 8341–8349. 11112773 10.1074/jbc.M007116200

[15] Chadee DN, Yuasa T, Kyriakis JM (2002) Direct activation of mitogen-activated protein kinase kinase kinase MEKK1 by the Ste20p homologue GCK and the adapter protein TRAF2. Mol Cell Biol 22: 737–749. 11784851 10.1128/MCB.22.3.737-749.2002 PMC133545

[16] Tsou HK, Su CM, Chen HT, Hsieh MH, Lin CJ, et al (2010) Integrin-linked kinase is involved in TNF-alpha-induced inducible nitric-oxide synthase expression in myoblasts. J Cell Biochem 109: 1244–1253. 20135642 10.1002/jcb.22508

[17] Shinoda S, Skradski SL, Araki T, Schindler CK, Meller R, et al (2003) Formation of a tumour necrosis factor receptor 1 molecular scaffolding complex and activation of apoptosis signal-regulating kinase 1 during seizure-induced neuronal death. Eur J Neurosci 17: 2065–2076. 12786973 10.1046/j.1460-9568.2003.02655.x

[18] Chang L, Kamata H, Solinas G, Luo JL, Maeda S, et al (2006) The E3 ubiquitin ligase itch couples JNK activation to TNFalpha-induced cell death by inducing c-FLIP(L) turnover. Cell 124: 601–613. 16469705 10.1016/j.cell.2006.01.021

[19] Karin M, Ben-Neriah Y (2000) Phosphorylation meets ubiquitination: the control of NF-[kappa]B activity. Annu Rev Immunol 18: 621–663. 10837071 10.1146/annurev.immunol.18.1.621

[20] Reuter S, Charlet J, Juncker T, Teiten MH, Dicato M, et al (2009) Effect of curcumin on nuclear factor kappaB signaling pathways in human chronic myelogenous K562 leukemia cells. Ann N Y Acad Sci 1171: 436–447. 19723087 10.1111/j.1749-6632.2009.04731.x

[21] Sugatani T, Hruska KA (2005) Akt1/Akt2 and mammalian target of rapamycin/Bim play critical roles in osteoclast differentiation and survival, respectively, whereas Akt is dispensable for cell survival in isolated osteoclast precursors. J Biol Chem 280: 3583–3589. 15545269 10.1074/jbc.M410480200

[22] Yin MJ, Christerson LB, Yamamoto Y, Kwak YT, Xu S, et al (1998) HTLV-I Tax protein binds to MEKK1 to stimulate IkappaB kinase activity and NF-kappaB activation. Cell 93: 875–884. 9630230 10.1016/s0092-8674(00)81447-6

[23] Pahl HL (1999) Activators and target genes of Rel/NF-kappaB transcription factors. Oncogene 18: 6853–6866. 10602461 10.1038/sj.onc.1203239

[24] Paz-Priel I, Houng S, Dooher J, Friedman AD C/EBPalpha and C/EBPalpha oncoproteins regulate nfkb1 and displace histone deacetylases from NF-kappaB p50 homodimers to induce NF-kappaB target genes. Blood 117: 4085–4094. 21346255 10.1182/blood-2010-07-294470 PMC3087533

[25] Subramanian A, Tamayo P, Mootha VK, Mukherjee S, Ebert BL, et al (2005) Gene set enrichment analysis: a knowledge-based approach for interpreting genome-wide expression profiles. Proc Natl Acad Sci U S A 102: 15545–15550. 16199517 10.1073/pnas.0506580102 PMC1239896

[26] Yang C, Aye CC, Li X, Diaz Ramos A, Zorzano A, et al (2012) Mitochondrial dysfunction in insulin resistance: differential contributions of chronic insulin and saturated fatty acid exposure in muscle cells. Biosci Rep 10.1042/BSR20120034 PMC3475448 22742515

[27] Baumgartner BG, Orpinell M, Duran J, Ribas V, Burghardt HE, et al (2007) Identification of a novel modulator of thyroid hormone receptor-mediated action. PLoS ONE 2: e1183 doi:10.1371/journal.pone.0001183. 18030323 10.1371/journal.pone.0001183 PMC2065906

[28] Francis VA, Zorzano A, Teleman AA (2010) dDOR is an EcR coactivator that forms a feed-forward loop connecting insulin and ecdysone signaling. Curr Biol 20: 1799–1808. 20888228 10.1016/j.cub.2010.08.055

[29] Lin J, Puigserver P, Donovan J, Tarr P, Spiegelman BM (2002) Peroxisome proliferator-activated receptor gamma coactivator 1beta (PGC-1beta), a novel PGC-1-related transcription coactivator associated with host cell factor. J Biol Chem 277: 1645–1648. 11733490 10.1074/jbc.C100631200

[30] Meirhaeghe A, Crowley V, Lenaghan C, Lelliott C, Green K, et al (2003) Characterization of the human, mouse and rat PGC1 beta (peroxisome-proliferator-activated receptor-gamma co-activator 1 beta) gene in vitro and in vivo. Biochem J 373: 155–165. 12678921 10.1042/BJ20030200 PMC1223480

[31] Lelliott CJ, Medina-Gomez G, Petrovic N, Kis A, Feldmann HM, et al (2006) Ablation of PGC-1beta results in defective mitochondrial activity, thermogenesis, hepatic function, and cardiac performance. PLoS Biol 4: e369 doi:10.1371/journal.pbio.0040369. 17090215 10.1371/journal.pbio.0040369 PMC1634886

[32] Sonoda J, Mehl IR, Chong LW, Nofsinger RR, Evans RM (2007) PGC-1beta controls mitochondrial metabolism to modulate circadian activity, adaptive thermogenesis, and hepatic steatosis. Proc Natl Acad Sci U S A 104: 5223–5228. 17360356 10.1073/pnas.0611623104 PMC1829290

[33] Hindorff LA, Sethupathy P, Junkins HA, Ramos EM, Mehta JP, et al (2009) Potential etiologic and functional implications of genome-wide association loci for human diseases and traits. Proc Natl Acad Sci U S A 106: 9362–9367. 19474294 10.1073/pnas.0903103106 PMC2687147

[34] Voight BF, Scott LJ, Steinthorsdottir V, Morris AP, Dina C, et al (2010) Twelve type 2 diabetes susceptibility loci identified through large-scale association analysis. Nat Genet 42: 579–589. 20581827 10.1038/ng.609 PMC3080658

[35] Segre AV, Groop L, Mootha VK, Daly MJ, Altshuler D (2010) Common inherited variation in mitochondrial genes is not enriched for associations with type 2 diabetes or related glycemic traits. PLoS Genet 6 doi:10.1371/journal.pgen.1001058. 10.1371/journal.pgen.1001058 PMC2920848 20714348

[36] Zeggini E, Weedon MN, Lindgren CM, Frayling TM, Elliott KS, et al (2007) Replication of genome-wide association signals in UK samples reveals risk loci for type 2 diabetes. Science 316: 1336–1341. 17463249 10.1126/science.1142364 PMC3772310

[37] Zeggini E, Scott LJ, Saxena R, Voight BF, Marchini JL, et al (2008) Meta-analysis of genome-wide association data and large-scale replication identifies additional susceptibility loci for type 2 diabetes. Nat Genet 40: 638–645. 18372903 10.1038/ng.120 PMC2672416

[38] Strawbridge RJ, Dupuis J, Prokopenko I, Barker A, Ahlqvist E, et al (2011) Genome-wide association identifies nine common variants associated with fasting proinsulin levels and provides new insights into the pathophysiology of type 2 diabetes. Diabetes 60: 2624–2634. 21873549 10.2337/db11-0415 PMC3178302

[39] Soranzo N, Sanna S, Wheeler E, Gieger C, Radke D, et al (2010) Common variants at 10 genomic loci influence hemoglobin A(C) levels via glycemic and nonglycemic pathways. Diabetes 59: 3229–3239. 20858683 10.2337/db10-0502 PMC2992787

[40] Saxena R, Hivert MF, Langenberg C, Tanaka T, Pankow JS, et al (2010) Genetic variation in GIPR influences the glucose and insulin responses to an oral glucose challenge. Nat Genet 42: 142–148. 20081857 10.1038/ng.521 PMC2922003

[41] Prokopenko I, Langenberg C, Florez JC, Saxena R, Soranzo N, et al (2009) Variants in MTNR1B influence fasting glucose levels. Nat Genet 41: 77–81. 19060907 10.1038/ng.290 PMC2682768

[42] Dupuis J, Langenberg C, Prokopenko I, Saxena R, Soranzo N, et al (2010) New genetic loci implicated in fasting glucose homeostasis and their impact on type 2 diabetes risk. Nat Genet 42: 105–116. 20081858 10.1038/ng.520 PMC3018764

[43] International Consortium for Blood Pressure Genome-Wide (2011) Association S, Ehret GB, Munroe PB, Rice KM, Bochud M, et al (2011) Genetic variants in novel pathways influence blood pressure and cardiovascular disease risk. Nature 478: 103–109. 21909115 10.1038/nature10405 PMC3340926

[44] Speliotes EK, Willer CJ, Berndt SI, Monda KL, Thorleifsson G, et al (2010) Association analyses of 249,796 individuals reveal 18 new loci associated with body mass index. Nat Genet 42: 937–948. 20935630 10.1038/ng.686 PMC3014648

[45] Heid IM, Jackson AU, Randall JC, Winkler TW, Qi L, et al (2010) Meta-analysis identifies 13 new loci associated with waist-hip ratio and reveals sexual dimorphism in the genetic basis of fat distribution. Nat Genet 42: 949–960. 20935629 10.1038/ng.685 PMC3000924

[46] Chambers JC, Zhang W, Sehmi J, Li X, Wass MN, et al (2011) Genome-wide association study identifies loci influencing concentrations of liver enzymes in plasma. Nat Genet 43: 1131–1138. 22001757 10.1038/ng.970 PMC3482372

[47] Patti ME, Butte AJ, Crunkhorn S, Cusi K, Berria R, et al (2003) Coordinated reduction of genes of oxidative metabolism in humans with insulin resistance and diabetes: Potential role of PGC1 and NRF1. Proc Natl Acad Sci U S A 100: 8466–8471. 12832613 10.1073/pnas.1032913100 PMC166252

[48] Mootha VK, Lindgren CM, Eriksson KF, Subramanian A, Sihag S, et al (2003) PGC-1alpha-responsive genes involved in oxidative phosphorylation are coordinately downregulated in human diabetes. Nat Genet 34: 267–273. 12808457 10.1038/ng1180

[49] Mootha VK, Handschin C, Arlow D, Xie X, St Pierre J, et al (2004) Erralpha and Gabpa/b specify PGC-1alpha-dependent oxidative phosphorylation gene expression that is altered in diabetic muscle. Proc Natl Acad Sci U S A 101: 6570–6575. 15100410 10.1073/pnas.0401401101 PMC404086

[50] Yadav H, Quijano C, Kamaraju AK, Gavrilova O, Malek R, et al (2011) Protection from obesity and diabetes by blockade of TGF-beta/Smad3 signaling. Cell Metab 14: 67–79. 21723505 10.1016/j.cmet.2011.04.013 PMC3169298

[51] Samani NJ, Erdmann J, Hall AS, Hengstenberg C, Mangino M, et al (2007) Genomewide association analysis of coronary artery disease. N Engl J Med 357: 443–453. 17634449 10.1056/NEJMoa072366 PMC2719290

[52] Ramos RR, Swanson AJ, Bass J (2007) Calreticulin and Hsp90 stabilize the human insulin receptor and promote its mobility in the endoplasmic reticulum. Proc Natl Acad Sci U S A 104: 10470–10475. 17563366 10.1073/pnas.0701114104 PMC1965537

[53] Sawa T, Imamura T, Haruta T, Sasaoka T, Ishiki M, et al (1996) Hsp70 family molecular chaperones and mutant insulin receptor: differential binding specificities of BiP and Hsp70/Hsc70 determines accumulation or degradation of insulin receptor. Biochem Biophys Res Commun 218: 449–453. 8561776 10.1006/bbrc.1996.0080

[54] Stranger BE, Nica AC, Forrest MS, Dimas A, Bird CP, et al (2007) Population genomics of human gene expression. Nat Genet 39: 1217–1224. 17873874 10.1038/ng2142 PMC2683249

[55] Nica AC, Parts L, Glass D, Nisbet J, Barrett A, et al (2011) The architecture of gene regulatory variation across multiple human tissues: the MuTHER study. PLoS Genet 7: e1002003 doi:10.1371/journal.pgen.1002003. 21304890 10.1371/journal.pgen.1002003 PMC3033383

[56] Yang J, Ferreira T, Morris AP, Medland SE (2012) Genetic Investigation of ATC, (2012) et al Conditional and joint multiple-SNP analysis of GWAS summary statistics identifies additional variants influencing complex traits. Nat Genet 44: 369–375. 22426310 10.1038/ng.2213 PMC3593158

[57] Pathmanathan S, Hamilton E, Atcheson E, Timson DJ (2011) The interaction of IQGAPs with calmodulin-like proteins. Biochem Soc Trans 39: 694–699. 21428964 10.1042/BST0390694

[58] White CD, Brown MD, Sacks DB (2009) IQGAPs in cancer: a family of scaffold proteins underlying tumorigenesis. FEBS Lett 583: 1817–1824. 19433088 10.1016/j.febslet.2009.05.007 PMC2743239

[59] Tekletsadik YK, Sonn R, Osman MA (2012) A conserved role of IQGAP1 in regulating TOR complex 1. J Cell Sci 125: 2041–2052. 22328503 10.1242/jcs.098947 PMC3360921

[60] Nauert JB, Rigas JD, Lester LB (2003) Identification of an IQGAP1/AKAP79 complex in beta-cells. J Cell Biochem 90: 97–108. 12938160 10.1002/jcb.10604

[61] Kaddai V, Gonzalez T, Keslair F, Gremeaux T, Bonnafous S, et al (2009) Rab4b is a small GTPase involved in the control of the glucose transporter GLUT4 localization in adipocyte. PLoS ONE 4: e5257 doi:10.1371/journal.pone.0005257. 19590752 10.1371/journal.pone.0005257 PMC2707114

[62] Sack MN (2010) Rab4a signaling unmasks a pivotal link between myocardial homeostasis and metabolic remodeling in the diabetic heart. J Mol Cell Cardiol 49: 908–910. 20840849 10.1016/j.yjmcc.2010.09.002 PMC2975788

[63] Barrett JC, Clayton DG, Concannon P, Akolkar B, Cooper JD, et al (2009) Genome-wide association study and meta-analysis find that over 40 loci affect risk of type 1 diabetes. Nat Genet 41: 703–707. 19430480 10.1038/ng.381 PMC2889014

[64] Schaefer CF, Anthony K, Krupa S, Buchoff J, Day M, et al (2009) PID: the Pathway Interaction Database. Nucleic Acids Res 37: D674–679. 18832364 10.1093/nar/gkn653 PMC2686461

[65] Planet E (2010) phenoTest: Tools to test correlation between gene expression and phenotype. Bioconductor R package version 1.0.0.

