# Supplementary material for: Identification of Novel Type 2 Diabetes Candidate Genes Involved in the Crosstalk between the Mitochondrial and the Insulin Signaling Systems
Source: PLoS Genet. 2012 Dec 6;8(12):e1003046. doi: 10.1371/journal.pgen.1003046 (PMC3516534; doi:10.1371/journal.pgen.1003046)
Supplement: Appendix S1 — List of authors and affiliations of the MITIN and the DIAGRAM+ consortia. (DOC) [file pgen.1003046.s001.doc]

**Appendix S1**

**List of authors and affiliations DIAGRAM+ consortium**

Benjamin F Voight ^1–3,100^, Laura J Scott ^4,100^, Valgerdur Steinthorsdottir ^5,100^, Andrew P Morris ^6,100^, Christian Dina ^7,8,100^, Ryan P Welch ^9^, Eleftheria Zeggini ^6,10^, Cornelia Huth ^11,12^, Yurii S Aulchenko ^13^, Gudmar Thorleifsson ^5^, Laura J Mcculloch ^14^, Teresa Ferreira ^6^, Harald Grallert ^11,12^, Najaf Amin ^13^, Guanming Wu ^15^, Cristen J Willer ^4^, Soumya Raychaudhuri ^1,2,16^, Steve A Mccarroll ^1,17^, Claudia Langenberg ^18^, Oliver M Hofmann ^19^, Josée Dupuis ^20,21^, Lu Qi ^22–24^, Ayellet V Segrè ^1,2,17^, Mandy van Hoek ^25^, Pau Navarro ^26^, Kristin Ardlie ^1^, Beverley Balkau ^27,28^, Rafn Benediktsson ^29,30^, Amanda J Bennett ^14^, Roza Blagieva ^31^, Eric Boerwinkle ^32^, Lori L Bonnycastle ^33^, Kristina Bengtsson Boström ^34^, Bert Bravenboer ^35^, Suzannah Bumpstead ^10^, Noisël P Burtt ^1^, Guillaume Charpentier ^36^, Peter S Chines ^33^, Marilyn Cornelis ^24^, David J Couper ^37^, Gabe Crawford ^1^, Alex S F Doney ^38,39^, Katherine S Elliott ^6^, Amanda L Elliott ^1,17,40^, Michael R Erdos ^33^, Caroline S Fox ^21,41^, Christopher S Franklin ^42^, Martha Ganser ^4^, Christian Gieger11, Niels Grarup ^43^, Todd Green ^1,2^, Simon Griffin ^18^, Christopher J Groves ^14^, Candace Guiducci ^1^, Samy Hadjadj ^44^, Neelam Hassanali ^14^, Christian Herder ^45^, Bo Isomaa ^46,47^, Anne U Jackson ^4^, Paul R V Johnson ^48^, Torben Jørgensen ^49,50^, Wen H L Kao ^51,52^, Norman Klopp ^11^, Augustine Kong ^5^, Peter Kraft ^22,23^, Johanna Kuusisto ^53^, Torsten Lauritzen ^54^, Man Li ^51^, Aloysius Lieverse ^55^, Cecilia M Lindgren ^6^, Valeriya Lyssenko ^56^, Michel Marre ^57,58^, Thomas Meitinger ^59,60^, Kristian Midthjell ^61^, Mario A Morken ^33^, Narisu Narisu ^33^, Peter Nilsson ^56^, Katharine R Owen ^14^, Felicity Payne ^10^, John R B Perry ^62,63^, Ann-Kristin Petersen ^11^, Carl Platou ^61^, Christine Proença ^7^, Inga Prokopenko ^6,14^, Wolfgang Rathmann ^64^, N William Rayner ^6,14^, Neil R Robertson ^6,14^, Ghislain Rocheleau ^65–67^, Michael Roden ^45,68^, Michael J Sampson ^69^, Richa Saxena ^1,2,40^, Beverley M Shields ^62,63^, Peter Shrader ^3,70^, Gunnar Sigurdsson ^29,30^, Thomas Sparsø ^43^, Klaus Strassburger ^64^, Heather M Stringham ^4^, Qi Sun ^22,23^, Amy J Swift ^33^, Barbara Thorand ^11^, Jean Tichet ^71^, Tiinamaija Tuomi ^46,72^, Rob M van Dam ^24^, Timon W van Haeften ^73^, Thijs van Herpt ^25,55^, Jana V van Vliet-Ostaptchouk ^74^, G Bragi Walters ^5^, Michael N Weedon ^62,63^, Cisca Wijmenga ^75^, Jacqueline Witteman ^13^, the mAgIc investigators ^99^, the gIAnt consortium ^99^, Richard N Bergman ^76^, Stephane Cauchi ^7^, Francis S Collins ^77^, Anna L Gloyn ^14^, Ulf Gyllensten ^78^, Torben Hansen ^43,79^, Winston A Hide ^19^, Graham A Hitman ^80^, Albert Hofman ^13^, David J Hunter ^22,23^, Kristian Hveem ^61,81^, Markku laakso ^53^, Karen L Mohlke ^82^, Andrew D Morris ^38,39^, Colin N A Palmer ^38,39^, Peter P Pramstaller ^83^, Igor Rudan ^42,84,85^, Eric Sijbrands ^25^, Lincoln D Stein ^15^, Jaakko Tuomilehto ^86–88^, Andre Uitterlinden ^25^, Mark Walker ^89^, Nicholas J Wareham ^18^, Richard M Watanabe ^76,90^, Gonçalo R Abecasis ^4^, Bernhard O Boehm ^31^, Harry Campbell ^42^, Mark J Daly ^1,2^, Andrew T Hattersley ^62,63^, Frank B Hu ^22–24^, James B Meigs ^3,70^, James S Pankow ^91^, Oluf Pedersen ^43,92,93^, H-Erich Wichmann ^11,12,94^, Inês Barroso ^10^, Jose C Florez ^1–3,95^, Timothy M Frayling ^62,63^, Leif Groop ^56,72^, Rob Sladek ^65–67^, Unnur Thorsteinsdottir ^5,96^, James F Wilson ^42^, Thomas Illig ^11^, Philippe Froguel ^17,97^, Cornelia M van duijn ^13^, Kari Stefansson 5,96, David Altshuler 1–3,17,40,95, Michael Boehnke ^4^ & Mark I Mccarthy ^6,14,98^

1 Broad Institute of Harvard and Massachusetts Institute of Technology (MIT), Cambridge, Massachusetts, USA.

2 Center for Human Genetic Research, Massachusetts General Hospital, Boston, Massachusetts, USA.

3 Department of Medicine, Harvard Medical School, Boston, Massachusetts, USA.

4 Department of Biostatistics, University of Michigan, Ann Arbor, Michigan, USA.

5 deCODE Genetics, Reykjavik, Iceland.

6 Wellcome Trust Centre for Human Genetics, University of Oxford, Oxford, UK.

7 CNRS-UMR-8090, Institute of Biology and Lille 2 University, Pasteur Institute, Lille, France.

8 INSERM UMR915 CNRS ERL3147, Nantes, France.

9 Bioinformatics Program, University of Michigan, Ann Arbor, Michigan, USA.

10 Wellcome Trust Sanger Institute, Hinxton, UK.

11 Institute of Epidemiology, Helmholtz Zentrum Muenchen, Neuherberg, Germany.

12 Institute of Medical Informatics, Biometry and Epidemiology, Ludwig-Maximilians-Universität, Munich, Germany.

13 Department of Epidemiology, Erasmus University Medical Center, Rotterdam, The Netherlands.

14 Oxford Centre for Diabetes, Endocrinology and Metabolism, University of Oxford, Oxford, UK.

15 Ontario Institute for Cancer Research, Toronto, Ontario, Canada.

16 Division of Rheumatology, Immunology and Allergy, Brigham and Women’s Hospital, Harvard Medical School, Boston, Massachusetts, USA.

17 Department of Molecular Biology, Harvard Medical School, Boston, Massachusetts, USA.

18 Medical Research Council (MRC) Epidemiology Unit, Institute of Metabolic Science, Addenbrooke’s Hospital, Cambridge, UK.

19 Department of Biostatistics, Harvard School of Public Health, Boston, Massachusetts, USA.

20 Department of Biostatistics, Boston University School of Public Health, Boston, Massachusetts, USA.

21 National Heart, Lung, and Blood Institute’s Framingham Heart Study, Framingham, Massachusetts, USA.

22 Department of Nutrition, Harvard School of Public Health, Boston, Massachusetts, USA.

23 Department of Epidemiology, Harvard School of Public Health, Boston, Massachusetts, USA.

24 Channing Laboratory, Department of Medicine, Brigham and Women’s Hospital and Harvard Medical School, Boston, Massachusetts, USA.

25 Department of Internal Medicine, Erasmus University Medical Centre, Rotterdam, The Netherlands.

26 MRC Human Genetics Unit, Institute of Genetics and Molecular Medicine, Western General Hospital, Edinburgh, UK.

27 INSERM, CESP Centre for Research in Epidemiology and Population Health, U1018, Epidemiology of Diabetes, Obesity and Chronic Kidney Disease over the Lifecourse, Villejuif, France.

28 University Paris-Sud 11, UMRS 1018, Villejuif, France.

29 Landspitali University Hospital, Reykjavik, Iceland.

30 Icelandic Heart Association, Kopavogur, Iceland.

31 Division of Endocrinology, Diabetes and Metabolism, Ulm University, Ulm, Germany.

32 The Human Genetics Center and Institute of Molecular Medicine, University of Texas Health Science Center, Houston, Texas, USA.

33 National Human Genome Research Institute, National Institute of Health, Bethesda, Maryland, USA.

34 Research and Development Centre, Skaraborg Primary Care, Skövde, Sweden.

35 Department of Internal Medicine, Catharina Hospital, Eindhoven, The Netherlands.

36 Endocrinology-Diabetology Unit, Corbeil-Essonnes Hospital, Corbeil-Essonnes, France.

37 Department of Biostatistics and Collaborative Studies Coordinating Center, University of North Carolina at Chapel Hill, Chapel Hill, North Carolina, USA.

38 Diabetes Research Centre, Biomedical Research Institute, University of Dundee, Ninewells Hospital, Dundee, UK.

39 Pharmacogenomics Centre, Biomedical Research Institute, University of Dundee, Ninewells Hospital, Dundee, UK.

40 Department of Genetics, Harvard Medical School, Boston, Massachusetts, USA.

41 Division of Endocrinology, Diabetes, and Hypertension, Brigham and Women’s Hospital, Harvard Medical School, Boston, Massachusetts, USA.

42 Centre for Population Health Sciences, University of Edinburgh, Edinburgh, UK.

43 Hagedorn Research Institute, Gentofte, Denmark.

44 Centre Hospitalier Universitaire de Poitiers, Endocrinologie Diabetologie, CIC INSERM 0801, INSERM U927, Université de Poitiers, UFR, Médecine Pharmacie, Poitiers Cedex, France.

45 Institute for Clinical Diabetology, German Diabetes Center, Leibniz Center for Diabetes Research at Heinrich Heine University Düsseldorf, Düsseldorf, Germany.

46 Folkhälsan Research Center, Helsinki, Finland.

47 Malmska Municipal Health Center and Hospital, Jakobstad, Finland.

48 Diabetes Research and Wellness Foundation Human Islet Isolation Facility and Oxford Islet Transplant Programme, University of Oxford, Oxford, UK.

49 Research Centre for Prevention and Health, Glostrup University Hospital, Glostrup, Denmark.

50 Faculty of Health Science, University of Copenhagen, Copenhagen, Denmark.

51 Department of Epidemiology, Johns Hopkins University, Baltimore, Maryland, USA.

52 Department of Medicine and Welch Center for Prevention, Epidemiology and Clinical Research, Johns Hopkins University, Baltimore, Maryland, USA.

53 Department of Medicine, University of Kuopio and Kuopio University Hospital, Kuopio, Finland.

54 Department of General Medical Practice, University of Aarhus, Aarhus, Denmark.

55 Department of Internal Medicine, Maxima Medical Center, Eindhoven, The Netherlands.

56 Department of Clinical Sciences, Diabetes and Endocrinology Research Unit, University Hospital Malmö, Lund University, Malmö, Sweden.

57 Department of Endocrinology, Diabetology and Nutrition, Bichat-Claude Bernard University Hospital, Assistance Publique des Hôpitaux de Paris, Paris, France.

58 INSERM U695, Université Paris 7, Paris, France.

59 Institute of Human Genetics, Helmholtz Zentrum Muenchen, Neuherberg, Germany.

60 Institute of Human Genetics, Klinikum rechts der Isar, Technische Universität München, München, Germany.

61 Nord-Trøndelag Health Study (HUNT) Research Center, Department of Community Medicine and General Practice, Norwegian University of Science and Technology, Trondheim, Norway.

62 Genetics of Complex Traits, Institute of Biomedical and Clinical Science, Peninsula Medical School, University of Exeter, Exeter, UK.

63 Diabetes Genetics, Institute of Biomedical and Clinical Science, Peninsula Medical School, University of Exeter, Exeter, UK.

64 Institute of Biometrics and Epidemiology, German Diabetes Center, Leibniz Center for Diabetes Research at Heinrich Heine University Düsseldorf, Düsseldorf, Germany.

65 Department of Human Genetics, McGill University, Montreal, Canada.

66 Department of Medicine, Faculty of Medicine, McGill University, Montreal, Canada.

67 McGill University and Genome Quebec Innovation Centre, Montreal, Canada.

68 Department of Metabolic Diseases, Heinrich Heine University Düsseldorf, Düsseldorf, Germany.

69 Department of Endocrinology and Diabetes, Norfolk and Norwich University Hospital National Health Service Trust, Norwich, UK.

70 General Medicine Division, Massachusetts General Hospital, Boston, Massachusetts, USA.

71 Institut interrégional pour la Santé (IRSA), La Riche, France.

72 Department of Medicine, Helsinki University Hospital, University of Helsinki, Helsinki, Finland.

73 Department of Internal Medicine, University Medical Center Utrecht, Utrecht, The Netherlands.

74 Molecular Genetics, Medical Biology Section, Department of Pathology and Medical Biology, University Medical Center Groningen and University of Groningen, Groningen, The Netherlands.

75 Department of Genetics, University Medical Center Groningen and University of Groningen, Groningen, The Netherlands.

76 Department of Physiology and Biophysics, University of Southern California School of Medicine, Los Angeles, California, USA.

77 National Institute of Health, Bethesda, Maryland, USA.

78 Department of Genetics and Pathology, Rudbeck Laboratory, Uppsala University, Uppsala, Sweden.

79 University of Southern Denmark, Odense, Denmark.

80 Centre for Diabetes, Barts and The London School of Medicine and Dentistry, Queen Mary University of London, London, UK.

81 Department of Medicine, The Hospital of Levanger, Levanger, Norway.

82 Department of Genetics, University of North Carolina, Chapel Hill, North Carolina, USA.

83 Institute of Genetic Medicine, European Academy Bozen/Bolzano (EURAC), Bolzano, Italy.

84 Croatian Centre for Global Health, Faculty of Medicine, University of Split, Split, Croatia.

85 Institute for Clinical Medical Research, University Hospital ‘Sestre Milosrdnice’, Zagreb, Croatia.

86 Department of Public Health, University of Helsinki, Helsinki, Finland.

87 South Ostrobothnia Central Hospital, Seinäjoki, Finland.

88 Red RECAVA Grupo RD06/0014/0015, Hospital Universitario La Paz, Madrid, Spain.

89 Diabetes Research Group, Institute of Cellular Medicine, Newcastle University, Newcastle upon Tyne, UK.

90 Department of Preventative Medicine, Keck Medical School, University of Southern California, Los Angeles, California, USA.

91 Division of Epidemiology and Community Health, University of Minnesota, Minneapolis, Minnesota, USA.

92 Department of Biomedical Science, Panum, Faculty of Health Science, University of Copenhagen, Copenhagen, Denmark.

93 Faculty of Health Science, University of Aarhus, Aarhus, Denmark.

94 Klinikum Grosshadern, Munich, Germany.

95 Diabetes Unit, Massachusetts General Hospital, Boston, Massachusetts, USA.

96 Faculty of Medicine, University of Iceland, Reykjavík, Iceland.

97 Genomic Medicine, Imperial College London, Hammersmith Hospital, London, UK.

98 Oxford National Institute for Health Research Biomedical Research Centre, Churchill Hospital, Oxford, UK.

99 A full list of members is provided in the supplementary Note of the original publication.

100 These authors contributed equally

**List of authors and affiliations MITIN consortium**

Josep M Mercader^1^, Montserrat Puiggros^1,2^, Eleonora M. Sorianello^3^, David Sebastian^3^, Sergio Rodriguez-Cuenca^4^, Chenjing Yang^5^, Sílvia Mora^5^, Antoni Vidal-Puig^4^, Matej Oresic^6^, Aurelio^7^ Teleman, Antonio Zorzano^3^, David Torrents^1,8^.

^1^Joint IRB-BSC program on Computational Biology. Barcelona Supercomputing Center, Barcelona, Catalonia, Spain

^2^Computational Bioinformatics. National Institute of Bioinformatics, Spain

^3^Institute for Research in Biomedicine (IRB Barcelona), Universitat de Barcelona, and CIBERDEM, Barcelona, 08028, Catalonia, Spain

^4^University of Cambridge, Metabolic Research Laboratories Institute of Metabolic Sciences, Box 289, Addenbrooke's Hospital Cambridge, CB2 0QQ United Kingdom.

^5^Institute of Translational Medicine, Cellular and Molecular Physiology, Liverpool, L69 3BX, United Kingdom

^6^VTT Technical Research Centre Of Finland (VTT), Finland.

^7^Aurelio Teleman, German Cancer Research Center (Dkfz), Germany

^8^Institució Catalana de Recerca i Estudis Avançats (ICREA) Pg. Lluís Companys 23, 08010 Barcelona, Spain
