# Supplementary material for: Identification of Novel Type 2 Diabetes Candidate Genes Involved in the Crosstalk between the Mitochondrial and the Insulin Signaling Systems
Source: PLoS Genet. 2012 Dec 6;8(12):e1003046. doi: 10.1371/journal.pgen.1003046 (PMC3516534; doi:10.1371/journal.pgen.1003046)
Supplement: Table S6 — SNPs with distance less than 250 Kb to the internode genes associated with complex diseases or traits related to type 2 diabetes. (DOC) [file pgen.1003046.s009.doc]

**Table S6. SNPs with distance less than 250 Kb to the intenode genes associated with complex diseases or traits related to type 2 diabetes.**

| **HGNC^1^** | **rsid** | **distance to associated SNP (bp)** | **disease_trait** | **pValue** | **OR_or_beta** | **Insulin Partners** | **Mitochondria Partners** |
| --- | --- | --- | --- | --- | --- | --- | --- |
| NFKBIB | rs472265 | 181204 | Type 2 diabetes | 9.00E-06 | 1.39 | AKT2; IKBKB | MTIF2; MTIF2 |
| RPS13 | rs11024074; rs381815 | 178717; 193668 | Diastolic blood pressure; Systolic blood pressure | 1.00E-06; 2.00E-09 | 0.33; 0.65 | EIF4E; NOLC1; RPS6 | TUFM; SLC25A5 |
| IGF2BP1 | rs46522 | 86177 | Coronary heart disease | 2.00E-08 | 1.06 | RPS6; NOLC1 | SLC25A5; TUFM |
| GNL3 | rs2590838; rs1108842; rs6784615 | 93086; 0; 208746; 208746 | Adiponectin levels; Adiponectin levels; Waist-hip ratio | 2.00E-13; 1.00E-13; 4.00E-10 | 0.03; 0.03; 0.04 | RPS6; NOLC1 | SLC25A5; TP53; TUFM |
| AR | rs5031002 | intronic | LDL cholesterol | 2.00E-07 | 0.3 | AKT1 | BRCA1 |
| SNORD58B | rs4939883; rs7241918; rs7241918; rs4939883; rs4939883; rs2156552; rs2156552; rs2156552; rs7240405; rs7228085 | 149115; 142854; 142854; 149115; 149115; 163569; 163569; 163569; 140991; 142715 | Cholesterol,total; Cholesterol,total; HDL cholesterol; HDL cholesterol; HDL cholesterol; HDL cholesterol; HDL cholesterol; HDL cholesterol; HDL cholesterol; Lipid metabolism phenotypes | 2.00E-11; 2.00E-19; 3.00E-49; 7.00E-15; 2.00E-11; 2.00E-07; 2.00E-12; 6.00E-12; 5.00E-10; 7.00E-11 | 0.07; 1.94; 1.31; 0.14; 0.1; 0.07; 0.03; 1.2; 2.27; 0.11 | RPS6; NOLC1 | TUFM; SLC25A5 |
| STAM | rs2437258 | 133909 | Cardiovascular disease risk factors | 3.00E-10 | 0.1 | PPP1CA | TIMM8A |
| RPL27A | rs4929949; rs10769908 | 99365; 219869 | Body mass index; Body mass index | 3.00E-09; 1.00E-06 | 0.06; NR | RPS6; NOLC1 | SLC25A5; TUFM |
| ITGB3 | rs7206971; rs7206971 | 3457; 3457 | Cholesterol,total; LDL cholesterol | 1.00E-08; 4.00E-09 | 1.01; 0.87 | PTK2; SRC | TGM2; TGM2 |
| RPLP0 | rs17431357 | 241396 | Insulin resistance/response | 1.00E-06 | 34 | RPS6; NOLC1 | SLC25A5; TUFM |
| SNAP23 | rs2412710 | 99644 | Triglycerides | 2.00E-08 | 7 | VAMP2; STX4 | VAMP1 |
| PUF60 | rs11136341; rs11136341 | 131514; 131514 | Cholesterol,total; LDL cholesterol | 9.00E-10; 4.00E-13 | 1.34; 1.4 | YWHAG; YWHAG | HSPD1; TRAP1 |
| ADA | rs1800961; rs1800961; rs1800961 | 205799; 205799; 205799 | Cholesterol,total; HDL cholesterol; HDL cholesterol | 6.00E-13; 8.00E-10; 1.00E-15 | 4.73; 0.19; 1.88 | GRB2 | NT5M; TYMP |
| DDX21 | rs1802295 | 186645 | Type 2 diabetes | 4.00E-08 | 1.08 | RPS6; NOLC1 | SLC25A5; TUFM |
| SNRPD2 | rs11671664; rs11671664; rs2287019; rs10423928 | 18435; 18435; 6729; 8409 | Body mass index; Body mass index; Body mass index; Two-hour glucose challenge |  |  | PPP1CA | HSPD1; TRAP1 |
| PLCG2 | rs2925979; rs2925979 | 237912; 237912 | Adiponectin levels; HDL cholesterol | 3.00E-21; 2.00E-11 |  | CBLB; PIK3CG; CALML3; GRB2 | PRKCA; PRKCA; PRKCA; PRKCA |
| POLH | rs998584; rs6905288; rs9472138; rs6905288 | 171195; 172172; 225061; 172172 | Adiponectin levels; Coronaryheartdisease; Type 2 diabetes; Waist-hip ratio | 3.00E-08; 7.00E-08; 4.00E-06; 2.00E-26 | 0.03; 1.23; 1.06; 0.05 | PKLR | POLG |
| RPL32 | rs2290159 | 247064 | Cholesterol,total | 4.00E-09 | 1.42 | RPS6; NOLC1 | TUFM; SLC25A5 |
| POLM | rs4607517; rs4607517; rs4607517; rs4607517; rs1799884; rs730497; rs3757840; rs1127065 | 113529; 113529; 113529; 113529; 106929; 101582; 109077; 137732 | Fasting glucose-related traits; Fasting glucose-related traits; Fasting plasma glucose; Fasting plasma glucose; Glycated hemoglobin levels; Glycated hemoglobin levels; Metabolic syndrome; Metabolic syndrome | 2.00E-16; 7.00E-92; 1.00E-07; 1.00E-25; 1.00E-20; 6.00E-12; 4.00E-13; 9.00E-11 | NR; NR; 1.15; 0.06; 0.04; 0.03; 0.1; 0.08 | PKLR | POLG |
| RPL6 | rs2074356 | 197593 | HDL cholesterol | 7.00E-37 | 0.04 | RPS6; NOLC1 | SLC25A5; TUFM |
| RPL21 | rs4771122 | 189352 | Body mass index | 9.00E-10 | 0.09 | RPS6; YWHAG; NOLC1 | TUFM; SLC25A5 |
| AK1 | rs7865146 | 9122 | Metabolic syndrome | 1.00E-06 | 1.19 | PKLR | NT5M |
| NCOA1 | rs713586; rs6545814 | 164437; 137745 | Body mass index; Body mass index | 6.00E-22; 1.00E-13 | 0.14; 3.26 | YWHAH | ESR1 |
| RPL7A | rs635634; rs579459; rs514659; rs635634 | 60069; 60901; 72866; 60069 | Cholesterol,total; Coronaryheartdisease; Coronaryheartdisease; LDL cholesterol | 9.00E-21; 4.00E-14; 8.00E-09; 8.00E-22 | 2.3; 1.1; 1.21; 2.05 | RPS6; NOLC1 | TUFM; SLC25A5 |
| RPS7 | rs11677370 | 212911 | Type 2 diabetes | 3.00E-06 | 1.35 | EIF4E; NOLC1; RPS6 | SLC25A5; TUFM |
| RASAL2 | rs516636; rs543874; rs10913469 | 207347; 173384; 149345 | Body mass index; Body mass index; Body mass index | 3.00E-09; 4.00E-23; 6.00E-08 | 0.05; 0.22; 3.36 | IKBKB; YWHAG | MTIF2; MTIF2 |
| WWOX | rs9923451; rs17797882 | intronic; 160354 | Obesity; Type 2 diabetes | 8.00E-07; 9.00E-07 | NR; 1.08 | MAPK8 | TP53 |
| TUBB1 | rs6015450; rs6015450 |  | Diastolic blood pressure; Systolic blood pressure | 6.00E-23; 4.00E-23 | 0.56; 0.9 | RPS6; NOLC1 | C1QBP; SLC25A5; TUFM |
| JUN | rs2811893 | 84317 | Diabetic retinopathy | 3.00E-07 | NR | MAPK3; MAPK8; MAPK10 | ESR1; BRCA1; BRCA1; BRCA1; ESR1; ESR1 |
| PLD1 | rs6794092 | 29604 | Body mass index | 2.00E-06 | 0.17 | CAV1 | PRKCA |
| NME3 | rs1065656 | 17105 | Insulin-like growth factors | 1.00E-11 | NR | PKLR | AK2; DUT; AK3L1; POLG |
| CCT5 | rs2607292; rs2967951 | 106094; 197583 | Body mass index; Body mass index | 4.00E-06; 1.00E-06 | 0.81; 0.77 | ACTA1 | IMMT |
| NFKB1 | rs13107325; rs13107325; rs13107325; rs13107325 | 233777; 233777; 233777; 233777 | Body mass index; Diastolic blood pressure; HDL cholesterol; Systolic blood pressure | 2.00E-13; 2.00E-17; 7.00E-11; 3.00E-14 | 0.19; 0.68; 0.84; 0.98 | IKBKB; SOCS3; MAP3K1; AKT2 | MTIF2; BCL2L1; BCL2L1; BCL2; MTIF2; MTIF2; BCL2; MTIF2 |
| HNRNPM | rs2967605; rs7255436 | 39913; 76455 | HDL cholesterol; HDL cholesterol | 1.00E-08; 3.00E-08 | 0.12; 0.45 | RPS6; NOLC1 | SLC25A5; TUFM |
| BIRC5 | rs4129767 | 182269 | HDL cholesterol | 5.00E-09 | 0.4 | RASA1 | DIABLO |
| LRPPRC | rs6756629; rs4299376; rs6544713; rs4299376; rs6756629 | 48273; 40787; 39482; 40787; 48273 | Cholesterol,total; Cholesterol,total; LDL cholesterol; LDL cholesterol; LDL cholesterol |  |  | IKBKB | MTIF2 |
| CREBBP | rs2444217 | 107660 | Body mass index | 9.00E-08 | NR | IKBKB; CREB1 | TP53 |
| RPS12 | rs7769051 | 8093 | Diabetic nephropathy | 2.00E-06 | 1.28 | RPS6; NOLC1 | SLC25A5; TUFM |
| POLR1D | rs4771122 | 174723 | Body mass index | 9.00E-10 | 0.09 | IKBKB | MTIF2 |
| MED1 | rs11869286 | 206335 | HDL cholesterol | 3.00E-14 | 0.51 | INPP5K; INPP5K | TP53; OPA1 |
| UBE2L3 | rs181362 | 0 | HDL cholesterol | 1.00E-08 | 0.46 | CBL | BRCA1 |
| RPL18 | rs492602 | 83624 | Cholesterol,total | 2.00E-10 | 1.27 | RPS6; NOLC1 | SLC25A5; TUFM |
| HIST1H1D | rs1800562; rs1800562; rs1799945; rs1800562; rs1800562; rs1799945; | 141299; 141299; 143261; 141299; 141299; 143261 | Cardiovascular disease risk factors; Cholesterol,total; Diastolic blood pressure; Glycated hemoglobin levels; LDL cholesterol; Systolic blood pressure | 5.00E-12; 2.00E-08; 2.00E-15; 3.00E-20; 6.00E-10; 8.00E-12 | 0.18; 2.16; 0.46; 0.06; 2.22; 0.63 | RPS6; NOLC1 | SLC25A5; TUFM |
| LYST | rs6429082 | 224212 | Adiposity | 3.00E-07 | NR | CALM1 | MRPL17 |
| POLK | rs2112347; rs12916; rs3846662; rs12916; rs3846662; rs12916; rs3846663; rs12654264; rs7703051 | 118273; 151042; 156497; 151042; 156497; 151042; 151855; 158978; 182094 | Body mass index; Cholesterol,total; Cholesterol,total; LDL cholesterol; LDL cholesterol; LDL cholesterol; LDL cholesterol; LDL cholesterol; LDL cholesterol | 2.00E-13; 9.00E-47; 3.00E-19; 1.00E-11; 2.00E-11; 5.00E-45; 8.00E-12; 1.00E-20; 1.00E-08 | 0.1; 2.84; 0.09; 0.02; 0.08; 2.45; 0.07; 0.1; 18 | PKLR | POLG |
| SMAD3* | rs17228212 | intronic | Coronaryheartdisease | 2.00E-07 | 1.21 | MAPK1; FOXO1; FOXO3 | CYP11A1; BRCA1 |
| FHL2 | rs6712932 | 136571 | Type 2 diabetes | 6.00E-06 | 1.52 | CREB1; FOXO1 | ESR1; TP53 |
| LRP1 | rs11613352; rs11613352 | 185446; 185446 | HDL cholesterol; Triglycerides | 2.00E-08; 4.00E-10 | 0.46; 2.7 | SRC; LRPAP1 | APBB1; APBB1 |
| ILF3 | rs11671653 | 35393 | Coronary heart disease | 9.00E-07 | 0.11 | RPS6; YWHAQ; NOLC1 | C1QBP; C1QBP; SLC25A5; TUFM; TUFM; SLC25A5; SLC25A5; TUFM |
| RPS9 | rs386000 | 39899 | HDL cholesterol | 4.00E-16 | 0.83 | RPS6; NOLC1 | SLC25A5; TOMM40; TUFM |
| TOP1 | rs6029526; rs6029526 | intronic; intronic | Cholesterol,total; LDL cholesterol | 3.00E-17; 3.00E-19 | 1.52; 1.41 | RPS6; NOLC1 | TUFM; SLC25A5; TP53 |
| CDK9 | rs7865146 | 66571 | Metabolic syndrome | 1.00E-06 | 1.19 | INPP5K; PPARGC1A | MRPS27 |
| RPS2 | rs1065656 | 173217 | Insulin-like growth factors | 1.00E-11 | NR | RPS6; NOLC1 | SLC25A5; TUFM |

*During the preparation of the manuscript, Yadav, et al. presented a model showing that Smad3 knock-out mice were protected from died-induced obesity and diabetes and that their adipocytes showed increased mitochondrial biogenesis with the corresponding increase in basal respiration .

^1^ HUGO Gene Nomenclature Committee (http://www.genenames.org/)
